# Dual blockades of TIM-3 and PD-1 effectively prevent hyper-progression and enhance the efficacy of anti-PD-1 therapy in high-grade serous ovarian cancer

**DOI:** 10.1038/s41419-025-08231-6

**Published:** 2025-11-28

**Authors:** Jie Li, Ying Zhou, Yahan Song, Geyang Dai, Xi Li, Yue Sun, Jing Wang, Rui Wei, Fei Li, Ling Xi

**Affiliations:** 1 Department of Obstetrics and Gynecology, National Clinical Research Center for Obstetrics and Gynecology, Tongji Hospital, Tongji Medical College, Huazhong University of Science and Technology, Wuhan, China; 2 Key Laboratory of Cancer Invasion and Metastasis (Ministry of Education), Hubei Key Laboratory of Tumor Invasion and Metastasis, Tongji Hospital, Tongji Medical College, Huazhong University of Science and Technology, Wuhan, China

**Keywords:** Tumour immunology, Immune evasion

## Abstract

The programmed cell death 1 (PD-1), exhibits limited efficacy in high-grade serous ovarian cancer (HGSOC), with an average response rate of 10 to 15%. Furthermore, hyper-progression disease (HPD), which mostly occurs under immune checkpoint blockade (ICB) therapy, is a potentially deleterious side effect of ICB therapy that accelerates disease progression in HGSOC patients. Our study aims to identify the approach to improve the efficacy of anti-PD-1 treatment on HGSOC in preclinical settings. The prominent TIM-3 upregulation in CD8^+^ tumor-infiltrating lymphocytes (TILs) and tumor-infiltrating dendritic cells (TIDCs) and the phenomenon of HPD were observed in ID8_VEGF_-bearing mice after anti-PD-1 treatment. TIM-3 and PD-1 co-blockades prevented the occurrence of HPD in pre-clinical models and prolonged their survival. Meanwhile, TIM-3 and PD-1 co-blockades effectively enhanced the function and proliferation of CD8^+^TILs and TIDCs from ID8_VEGF_-bearing mice. Notably, TIM-3 and PD-1 inhibitors effectively enhanced the anti-tumor immunity of CD8^+^TILs and CD11c^+^ myeloid cells from HGSOC patients. Our study uncovers the significance of TIM-3 inhibition in preventing the occurrence of HPD and enhancing the efficacy of anti-PD-1 therapy in HGSOC.

## Introduction

High-grade serous ovarian cancer (HGSOC) is the most prevalent histological subtype of ovarian cancer (OC), where 70–80% of patients relapse despite optimal surgery and first-line platinum/taxane chemotherapy [1, 2]. In recent decades, immunotherapy, particularly immune checkpoint blockade (ICB) therapy, has transformed the landscape of cancer treatment [3]. However, the clinical efficacy of ICB targeting programmed cell death protein 1 (PD-1) in HGSOC has been notably limited compared to other malignancies, partly due to the occurrence of adaptive resistance and hyper-progression disease (HPD) [4]. Notably, the objective response rates (ORR) for HGSOC patients treated with PD-1 inhibitors remain low, as demonstrated by rates of 7.4% and 9.9% in cohorts A and B, respectively, of the KEYNOTE-100 study and 11.5% in the KEYNOTE-028 study [5, 6]. Additionally, prior studies have reported that up to 16.7% of patients experience HPD during anti-PD-1 therapy, which is associated with significantly worse outcomes compared with conventional disease progression [7]. These findings highlight the urgent need for effective combination therapy to enhance clinical responses and prevent the occurrence of HPD in HGSOC.

T-cell immunoglobulin and mucin domain-containing-3 (TIM-3) has emerged as a promising therapeutic target in OC due to its prevalent expression in tumor tissues [8] and its strong correlation with the poor prognosis in HGSOC patients [9]. Initially identified on IFN-γ-secreting CD4^+^ and CD8^+^ cytotoxic T cells, TIM-3 is now widely recognized as the marker of T cell exhaustion [10]. The interaction of TIM-3 with its ligands, like galectin-9, promotes T-cell apoptosis, and the formation of TIM-3/galectin-9/PD-1 lattices helps the survival of TIM-3^+^T cells, leading to the immunosuppressive tumor microenvironment (TME) [11]. Despite its role in T cells, TIM-3 expressed on dendritic cells (DCs) has been shown to negatively regulate their function negatively. Loss of TIM-3 on DCs enhances the expansion of stem-like CD8^+^T cells, boosts their antigen-specific immunity, and thus increases the anti-tumor activity of CD8^+^ T cells [12, 13]. Furthermore, IL-12-producing DCs play a pivotal role in the success of anti-PD-1 therapy by promoting the expansion of CD8^+^T cells, and exhausted CD8^+^TILs that failed to respond to anti-PD-1 blockade will be rescued by the antigen-presenting cells (APCs) agonist in the ID8 tumor-bearing mouse model [14–16]. In HGSOC, TIM-3 is abundantly expressed on both CD8^+^T cells and CD4^+^T cells of tumors and ascites [17]. However, research on the impact of TIM-3-targeted therapy in HGSOC is rather limited. These gaps underscore the need for further studies to elucidate the therapeutic potential of targeting TIM-3 in HGSOC patients.

Combinational immunotherapy has demonstrated the potential to augment the clinical efficacy of ICB therapy. For example, the concurrent blockade of PD-1 and lymphocyte-activation gene 3 (LAG-3) has enhanced the cytotoxic function of CD8^+^T cells in melanoma patients, improving therapeutic outcomes [18, 19]. Similarly, recent preclinical studies highlight the therapeutic benefits of combining TIM-3 inhibitors with other ICB across various cancer types [20, 21]. Preliminary clinical trial data further suggest that the dual inhibition of TIM-3 and PD-1 may represent a promising therapeutic strategy for patients unresponsive to anti-PD-1 monotherapy [22]. Although this combination approach is still in the early stages of the investigation, it holds significant potential to improve the prognosis of HGSOC patients. Further research is warranted to validate the efficacy of TIM-3 and PD-1 co-targeted therapies and to elucidate the underlying mechanisms.

Based on the background above, we investigated the underlying mechanisms contributing to HPD, and evaluated the therapeutic efficacy of combination therapy with TIM-3 and PD-1 inhibitors through both ID8_VEGF_-bearing peritoneal metastasis model and subcutaneous tumor model, and further validated the ex vivo efficacy of TIM-3 and PD-1 inhibitors in CD8^+^TILs and CD11c^+^ myeloid cells from HGSOC patients.

## Results

### TIM-3 is upregulated on CD8^+^TILs and TIDCs of ID8_VEGF_-bearing mice after anti-PD-1 treatment

**Fig. 1:**
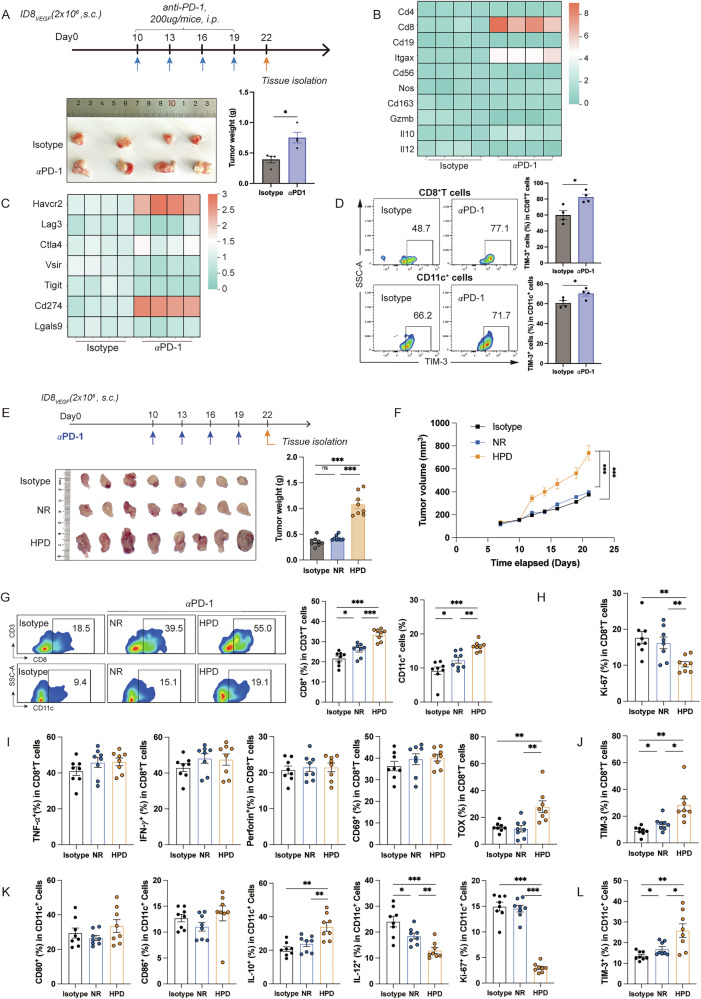
TIM-3 expression is increased on CD8^+^TILs and TIDCs of ID8_VEGF_-bearing mice with anti-PD-1 treatment. **A** The experimental arrangement of anti-PD-1 on ID8_VEGF_-bearing mice model, and the tumor weight on day 22. **B** The heatmap of mRNA expression of immune cell-related genes in tumors from ID8_VEGF_-bearing mice with anti-PD-1 treatment (n = 4 for each group). **C** The heatmap of mRNA expression of inhibitory immune checkpoint genes in tumors from ID8_VEGF_-bearing mice with anti-PD-1 treatment (n = 4 for each group). **D** Representative flow cytometry plots of TIM-3 expression in CD8^+^TILs and TIDCs and their percentages in ID8_VEGF_ tumor-bearing mice treated with isotype or anti-PD-1 (n = 4 for each group). **E** The experimental schedule of anti-PD-1 treatment in the ID8_VEGF_-bearing mice model, and the tumor weight on day 22. **F** Tumor growth curves of subcutaneous ID8_VEGF_ mice treated with isotype or anti-PD-1 (n = 10 for the isotype group, n = 12 for the NR group, n = 28 for the HPD group). **G** Representative flow cytometry plots and percentages of CD8^+^TILs and TIDCs from mice in isotype, NR, and HPD groups (n = 8 for each group). **H**, **I**, **J** Percentages of Ki-67, TNF-α, IFN-γ, Perforin, CD69, TOX, and TIM-3 expression in CD8^+^TILs from mice in isotype, NR, and HPD groups (n = 8 for each group). **K**, **L** Percentages of CD80, CD86, IL-10, IL-12, Ki-67, and TIM-3 expression in TIDCs from mice in isotype, NR, and HPD groups (n = 8 for each group). Bars represent mean ± SEM, and dots represent individual mice. *P < 0.05, **P < 0.01, ***P < 0.001.

Previous clinical studies have shown the limited efficacy of anti-PD-1 treatment in HGSOC patients. Similarly, in subcutaneous ID8_VEGF_-bearing mouse model, no obvious anti-tumor efficacy of anti-PD-1 therapy was observed. Notably, the obvious sudden tumor progression was observed in the subcutaneous ID8_VEGF_-bearing mouse model during anti-PD-1 therapy, which is defined as the HPD. HPD was characterized by rapid tumor growth and the tumor growth rate (TGR) ratio ≧2, which is consistent with the previous research [23]. Tumors from mice with HPD were isolated and displayed as in Fig. 1A. To explore the immune status change in TME, tumors isolated were analyzed using RT-qPCR, and results indicated that among all the immune cell-related genes, *Cd8* and *Itgax* were significantly regulated (Fig. 1B). Further analysis revealed that among all inhibitory immune checkpoint genes, *Havcr2* and *Cd274* mRNA expression levels were significantly increased (Fig. 1C). We also observed TIM-3 upregulation in CD8^+^TILs and TIDCs of ID8_VEGF_-bearing mice after anti-PD-1 treatment (Fig. 1D). These results uncovered that TIM-3 expression on CD8^+^TILs and TIDCs might have important roles in the HPD phenomenon. According to the previous studies [24], we established the preclinical ovarian tumor-bearing mouse model mimicking HPD induced by anti-PD-1 monotherapy as shown in Fig. 1E, F. Flow cytometry was used to explore the TME of mice with HPD, and the gating strategy was shown in Supplementary Fig. 1. Consistent with the results above, mice demonstrating HPD showed increased infiltration of CD8^+^TILs and TIDCs (Fig. 1G and Supplementary Fig. 2A), and significant upregulation of TIM-3 in CD8^+^TILs and TIDCs (Fig. 1J, L). Furthermore, the increased CD8^+^TILs infiltration in HPD mice showed decreased proliferation and increased TOX expression, with no obvious change in TNF-α, IFN-γ, Perforin, and CD69 expression (Fig. 1H, I). The TIDC from mice with HPD demonstrated increased IL-10 expression, decreased IL-12 secretion and proliferation, with no change in maturation (Fig. 1K). A significant decrease in Th1 cell infiltration was observed in the TME of mice with HPD, with no obvious change in the infiltration, proliferation, and TIM-3 expression (Supplementary Fig. 2A, B). The inhibitory immune cells, including Th2 cells, Tregs, and MDSCs, were highly infiltrated in the TME of mice with HPD, with enhanced proliferation levels and increased TIM-3 expression (Supplementary Fig. 2A, C–E). Furthermore, the infiltration, proliferation, IL-10 secretion, and TIM-3 expression were significantly increased in macrophages (Supplementary Fig. 2A, F). Collectively, the results above depict the increased infiltration and TIM-3 expression of CD8^+^T cells, TIDCs, and inhibitory immune cells in the TME of mice with HPD, which identify TIM-3 as a potential therapeutic target.

### TIM-3 and PD-1 co-blockades reverse the tumor progression induced by HPD in ID8_VEGF_-bearing mice during anti-PD-1 treatment by enhancing the function of CD8^+^TILs and TIDCs

**Fig. 2:**
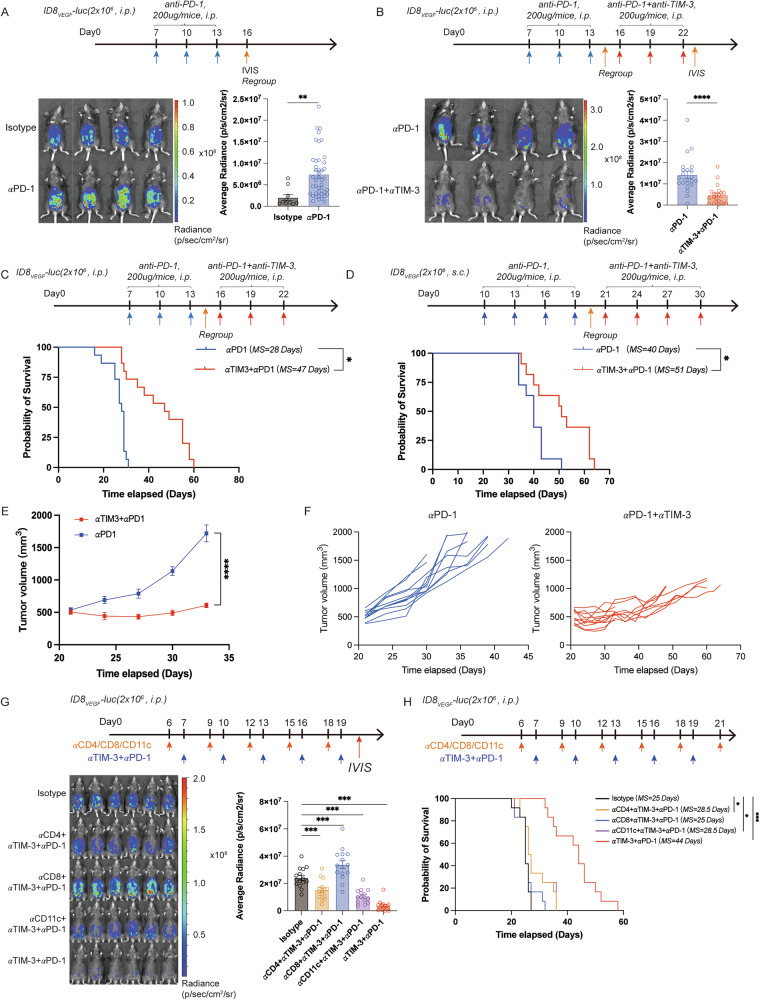
Anti-TIM-3 enhances the preclinical efficacy of anti-PD-1 treatment on ID8_VEGF_-bearing mice with HPD induced by anti-PD-1 therapy. **A** Experimental arrangements, the representative IVIS imaging, and average radiance of ID8_VEGF_-bearing mice model under anti-PD-1 treatment. **B** Representative IVIS imaging and average radiance of regrouped mice treated with anti-PD-1 or anti-PD-1 and anti-TIM-3. **C** The experimental arrangement and the survival curve of subcutaneous ID8_VEGF_ tumor-bearing mice (n = 11 for each group). **D** The experimental arrangement and the survival curve of ID8_VEGF_ tumor-bearing mice (n = 15 for each group). **E**, **F** Tumor growth curves of subcutaneous ID8_VEGF_-bearing mice demonstrating HPD treated with anti-PD-1 monotherapy or combination therapy with anti-PD-1 and anti-TIM-3 (n = 11 for each group). **G**, **H** The experimental arrangement, representative IVIS imaging, average radiance, and the survival curve of ID8_VEGF_-bearing mice treated with anti-PD-1 and anti-TIM-3 and depleting antibody specific for CD4^+^ T cells, CD8^+^ T cells, and CD11c^+^ cells (n = 12 for each group). Bars represent mean ± SEM, and dots represent individual mice. *P < 0.05, **P < 0.01, ***P < 0.001, ****P < 0.0001.

**Fig. 3:**
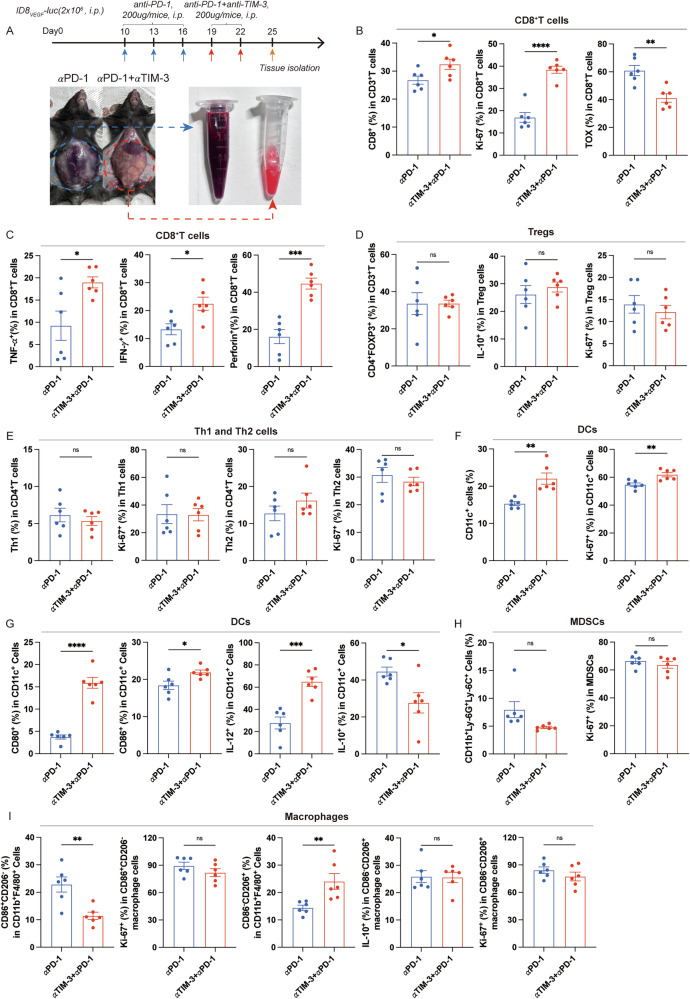
Anti-TIM-3 and anti-PD-1 combinational therapy enhances the anti-tumor immunity of CD8^+^TILs and TIDCs in ID8_VEGF_-bearing mice with HPD induced by anti-PD-1 treatment. **A** The experimental arrangement and the representative pictures of ascites from ID8_VEGF_-bearing mice demonstrating HPD. **B**, **C** Percentages of CD8^+^TILs and Ki-67, TOX, TNF-α, IFN-γ, and Perforin expression on CD8^+^TILs from ID8_VEGF_ tumor-bearing mice treated with anti-PD-1 or anti-PD-1 and anti-TIM-3 (n = 6 for each group). **D**, **E** Percentages of Tregs, IL-10, and Ki-67 expression in Tregs, Th1, Th2 cells, and Ki-67 expression in Th1, Th2 cells in tumors from ID8_VEGF_ tumor-bearing mice treated with anti-PD-1 or anti-PD-1 and anti-TIM-3 (n = 6 for each group). **F**, **G** Percentages of TIDCs and CD80, CD86, IL-12, IL-10, and Ki-67 expression on TIDCs from ID8_VEGF_ tumor-bearing mice treated with anti-PD-1 or anti-PD-1 and anti-TIM-3 (n = 6 for each group). **H** Percentages of MDSCs and their Ki-67 expression in tumors from ID8_VEGF_ tumor-bearing mice treated with anti-PD-1 or anti-PD-1 and anti-TIM-3 (n = 6 for each group). **I** Percentages of CD86^+^CD206^-^ macrophage cells, CD86^-^CD206^+^ macrophage cells, Ki-67 expression in CD86^+^CD206^-^ macrophage cells, IL-10, and Ki-67 expression in CD86^-^CD206^+^ macrophage cells in tumors from ID8_VEGF_ tumor-bearing mice treated with anti-PD-1 or anti-PD-1 and anti-TIM-3 (n = 6 for each group). Bars represent mean ± SEM, and dots represent individual mice. *P < 0.05, **P < 0.01, ***P < 0.001, ****P < 0.0001.

We then unravel the efficacy of TIM-3-targeted therapy in the ID8_VEGF_-bearing mouse model with HPD triggered by anti-PD-1 treatment. To evaluate the therapeutic efficacy of TIM-3 and PD-1 co-blockades, intraperitoneal and subcutaneous ID8_VEGF_-bearing mouse models with HPD during anti-PD-1 treatment were established, as described above. Similarly, the intraperitoneal ID8_VEGF_-bearing mice under anti-PD-1 therapy progressed faster than those with the isotype treatment (Fig. 2A). Then the ID8_VEGF_-bearing mice under anti-PD-1 therapy with a higher average radiance than those treated with the isotype were selected and randomly regrouped, followed by anti-PD-1 monotherapy or anti-PD-1 and anti-TIM-3 combinational therapy. TIM-3 and PD-1 co-blockades significantly inhibited tumor growth and prolonged the survival time of ID8_VEGF_-bearing mice with HPD during anti-PD-1 treatment compared to anti-PD-1 monotherapy (Fig. 2B, C). For the subcutaneous ID8_VEGF_-bearing mouse under anti-PD-1 treatment, those with a TGR ratio ≧2 were selected and regrouped, followed by anti-PD-1 monotherapy or anti-PD-1 and anti-TIM-3 combinational therapy. Results showed the obvious delay in tumor growth and increase in survival time in mice with HPD treated with anti-PD-1 and anti-TIM-3 (Fig. 2D–F). Data above indicated the preclinical efficacy of the combinational therapy of anti-PD-1 and anti-TIM-3 in reversing the rapid tumor progression caused by HPD. Considering that TIM-3 was upregulated in multiple immune cells, we depleted these cells to decipher their contribution to the therapeutic effect, and the depletion efficacy was displayed in Supplementary Fig. 3. Depletion of CD4^+^ T cells and CD11c^+^ cells resulted in a partial loss of the therapeutic efficacy of anti-PD-1 and anti-TIM-3 combinational therapy, but the depletion of CD8^+^ T cells led to complete loss of therapeutic efficacy (Fig. 2G, H). Next, we investigated the effects of anti-PD-1 and anti-TIM-3 combinational therapy in the TME of mice with HPD. The ascites from mice were collected at the end of therapy (Fig. 3A). TIM-3 and PD-1 co-blockades enhanced the infiltration of CD8^+^TILs, and improved the anti-tumor immunity and proliferation of CD8^+^TILs (Fig. 3B, C and Supplementary Fig. 4). Furthermore, the combination therapy increased the TIDCs infiltration and enhanced the proliferation, maturation, IL-12 expression, and decreased IL-10 expression in TIDCs from tumors of mice with HPD (Fig. 3F, G). Notably, no obvious effects of combination therapy were observed in the infiltration, proliferation, or function of Th1, Th2, Treg cells, and MDSCs (Fig. 3D, E, H, and Supplementary Fig. 4). Interestingly, the infiltration of macrophages in the TME of mice with combination therapy was increased, with a specific increase in frequency of CD86^-^CD206^+^ macrophage cells and a decrease in frequency of CD86^+^CD206^-^ macrophage cells (Fig. 3I and Supplementary Fig. 4). Collectively, these results indicated that the TIM-3 and PD-1 blockades reversed the immunosuppressive TME of mice with HPD, mediated by the activation of CD8^+^ T cells and TIDCs, which enhanced the anti-tumor immunity.

### TIM-3 and PD-1 co-blockades delay tumor growth and prolong the survival time of ID8_VEGF_ tumor-bearing mice

**Fig. 4:**
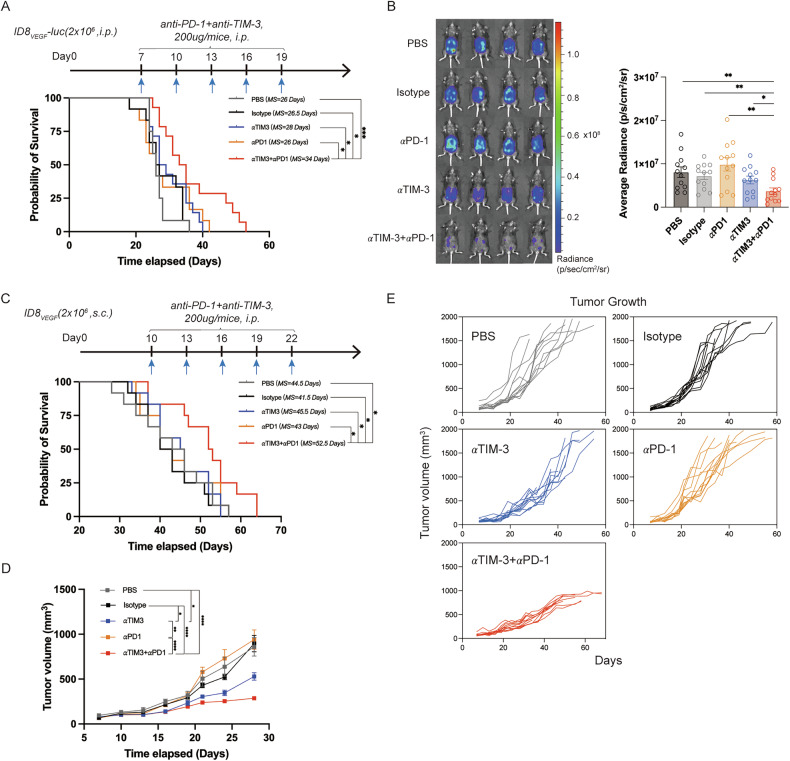
TIM-3 and PD-1 co-blockades delay tumor growth and prolong their survival time in ID8_VEGF_ tumor-bearing mice. **A** The experimental arrangement and the survival curve of subcutaneous ID8_VEGF_ tumor-bearing mice (n = 12 for each group). **B** The representative IVIS images and average radiance of ID8_VEGF_ tumor-bearing mice with different treatments (n = 12 for each group). **C** The experimental arrangement and the survival curve of ID8_VEGF_ tumor-bearing mice (n = 12 for each group). **D**, **E** Tumor growth and average tumor volume by days of subcutaneous ID8_VEGF_-bearing mice with different treatments (n = 12 for each group). Bars represent mean ± SEM, and dots represent individual mice. *P < 0.05, **P < 0.01, ***P < 0.001, ****P < 0.0001.

**Fig. 5:**
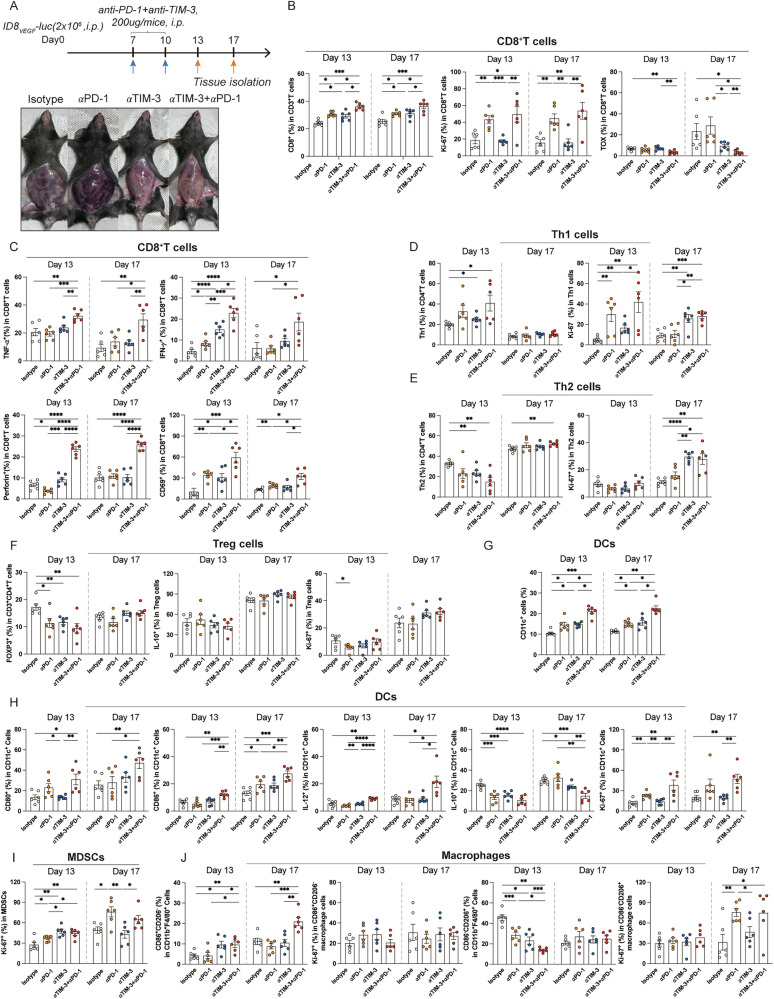
TIM-3 and PD-1 co-blockades effectively enhance the function and proliferation of CD8^+^TILs and TIDCs from ID8_VEGF_-bearing mice. **A** The experimental arrangement and images of ascites isolated from ID8_VEGF_ tumor-bearing mice on day 17. **B**, **C** Percentages of CD8^+^TILs, and IFN-γ, TNF-α, Perforin, CD69, Ki-67, and TOX expression on CD8^+^TILs from ID8_VEGF_ tumor-bearing mice with different treatments (n = 6 for each group). **D**–**F** Percentages of Th1, Th2 cells, Ki-67 expression in Th1, Th2 cells, Tregs, and IL-10 and Ki-67 expression in Tregs in tumors from ID8_VEGF_ tumor-bearing mice with different treatments (n = 6 for each group). **G**, **H** Percentages of TIDCs, and Ki-67, CD80, CD86, IL-12, and IL-10 expression on TIDCs from ID8_VEGF_ tumor-bearing mice with different treatments (n = 6 for each group). **I** Percentages of Ki-67 in MDSCs in tumors from ID8_VEGF_ tumor-bearing mice with different treatments (n = 6 for each group). **J** Percentages of CD86^+^CD206^-^ macrophage cells, CD86^-^CD206^+^ macrophage cells, and their Ki-67 expression in tumors from ID8_VEGF_ tumor-bearing mice with different treatments (n = 6 for each group). Bars represent mean ± SEM, and dots represent individual mice. *P < 0.05, **P < 0.01, ***P < 0.001, ****P < 0.0001.

The findings above showed the remarkable effects of TIM-3 and PD-1 blockades in preventing HPD in mice under anti-PD-1 monotherapy, and we further evaluated the in vivo preclinical applicative value of TIM-3 and PD-1 co-blockades in treating ovarian tumor-bearing mice. As shown in Fig. 4A, B, treatment with TIM-3 and PD-1 co-blockades could effectively delay tumor growth and prolong the survival time of intraperitoneal ID8_VEGF_-bearing mice. TIM-3 and PD-1 co-blockades showed similar efficacy in subcutaneous ID8_VEGF_-bearing mice, as the inhibition in tumor growth and extended survival time (Fig. 4C–E). Further analysis was conducted on the effects of TIM-3 and PD-1 co-blockades on the TME of intraperitoneal and subcutaneous ID8_VEGF_ tumor-bearing mice by flow cytometry, with experiment arrangements shown in Fig. 5A and Supplementary Fig. 6A. In intraperitoneal ID8_VEGF_ tumor-bearing mice, the proportion of CD8^+^TILs from mice treated with TIM-3 and PD-1 inhibitors combination therapy was increased and showed the most active proliferation, the lowest TOX expression, and the highest TNF-α, IFN-γ, Perforin, and CD69 expression, compared to single treatment or isotype (Fig. 5B, C and Supplementary Fig. 5). Furthermore, TIM-3 and PD-1 inhibitors increased the percentage of tumor-infiltrating Th1 cells while reducing Th2 cell proportions, and led to a noticeable decrease in tumor-infiltrating Tregs compared to isotype controls (Fig. 5D–F and Supplementary Fig. 5). The infiltration and proliferation of TIDCs were markedly increased compared to isotype controls or single treatments, and TIDCs exhibited the highest levels of maturity (CD80 and CD86 expression), IL-12 secretion, proliferation, along with the lowest IL-10 secretion, following combination therapy (Fig. 5G, H). MDSCs exhibited enhanced proliferation over time post-treatment, with no obvious change in total infiltration in TME (Fig. 5I and Supplementary Fig. 5). The combination therapy also significantly enhanced the infiltration and function of APCs. The infiltration of macrophages in TME was increased, with a specific increase in percentages of CD86^+^CD206^-^ macrophage cells, and a decrease in CD86^-^CD206^+^ macrophage cells (Fig. 5J and Supplementary Fig. 5). Consistent with the results above, analysis from subcutaneous ID8_VEGF_ tumor-bearing mice showed that the combination of TIM-3 and PD-1 inhibitors could enhance the infiltration, proliferation, and anti-tumor immunity of CD8^+^TILs (Supplementary Fig. 6B, C). The proportion, proliferation, maturation, and IL-12 expression of TIDCs were also enhanced, and IL-10 expression was decreased in TIDCs (Supplementary Fig. 6D, E).

### TIM-3 and PD-1 inhibitors combined therapy reverses the CD8^+^TILs and CD11c^+^ myeloid cells exhaustion ex vivo

**Fig. 6:**
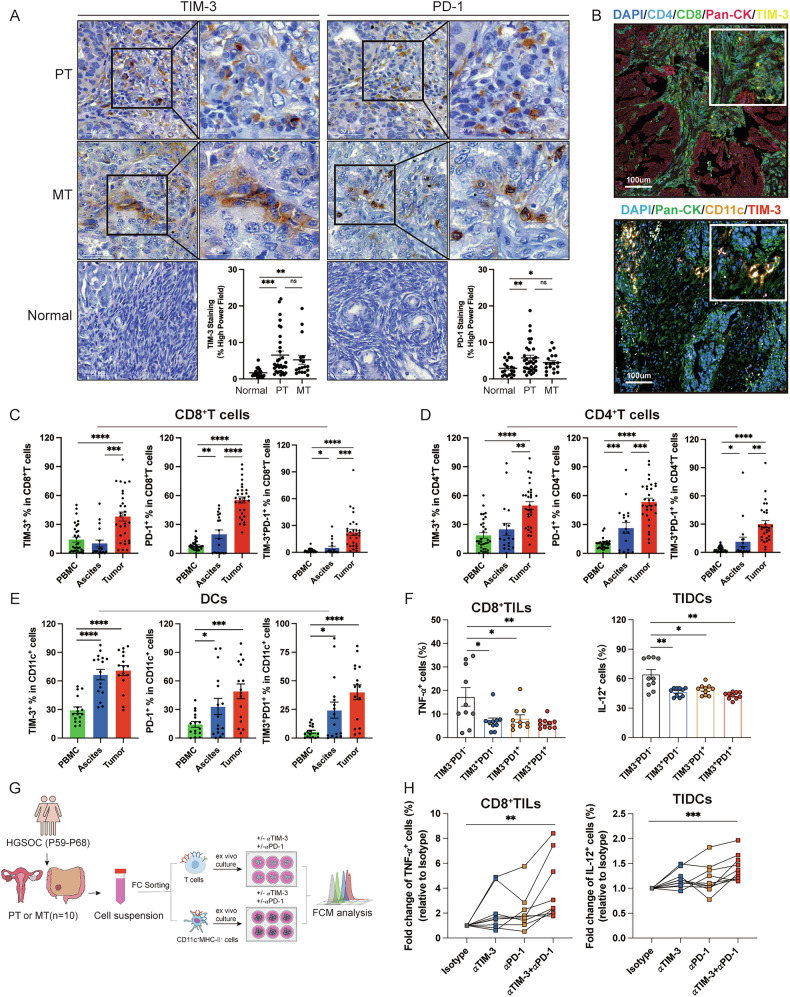
TIM-3 and PD-1 co-blockades enhance the function of CD8^+^TILs and CD11c^+^ myeloid cells from HGSOC patients ex vivo. **A** Representative IHC staining images of TIM-3 and PD-1 expression and their percentages in normal ovarian tissue (n = 24), primary tumors (PT) (n = 37), and metastatic tumors (MT) (n = 23). Scale bars are provided on each image. **B** Representative images of IF staining for CD4 (cyan), CD8 (green), pan-CK (red), and TIM-3 (yellow) in tumor tissues from HGSOC patients were shown on the left, and for TIM-3 (red), CD11c (orange), and pan-CK (green) were shown on the right. DNA was visualized with Hoechst 33342 (blue). Magnifications are given on the top right of each image, and scale bars are shown in each image. **C**, **D** Percentages of TIM-3 and PD-1 expression and co-expression on CD8^+^T cells and CD4^+^T cells from tumors (n = 30), ascites (n = 18), and blood (n = 32) of HGSOC patients. **E** Percentages of TIM-3 and PD-1 expression and co-expression on CD11c^+^ myeloid cells from tumors (n = 16), ascites (n = 16), and blood (n = 16) of HGSOC patients. **F** Percentages of TNF-α in CD8^+^TILs, IL-12 in CD11c^+^ myeloid cells of HGSOC patients (n = 10). **G** The experimental scheme for functional assay of CD8^+^TILs and CD11c^+^ myeloid cells from HGSOC patients (n = 10). **H** The fold change of TNF-α expression in CD8^+^TILs and IL-12 expression in CD11c^+^ myeloid cells isolated from HGSOC patients (n = 10) treated with anti-TIM-3 and anti-PD-1 blockade ex vivo, compared with those treated with Isotype. Bars represent mean ± SEM, and dots represent individual patients. *P < 0.05, **P < 0.01, ***P < 0.001, ****P < 0.0001.

The above results demonstrated the important role of TIM-3 in the development of HPD and the preclinical therapeutic effect of TIM-3 and PD-1 co-blockades. To further these findings in HGSOC patients, we explored the expression of inhibitory immune checkpoints in samples from HGSOC patients. Samples from HGSOC patients used in IHC and IF experiments were shown in Supplementary Fig. 7A. IHC results showed that among TIM-3, PD-1, LAG-3, cytotoxic T-lymphocyte antigen 4 (CTLA-4), T cell immunoreceptor with immunoglobulin and ITIM domain (TIGIT), and V-domain Ig suppressor of T cell activation (VISTA), TIM-3, PD-1, LAG-3, and VISTA were highly expressed in the stroma regions of primary or metastatic tumors of HGSOC patients, compared with normal ovarian tissues, with no noticeable difference observed in tumors in situ or metastasis (Fig. 6A and Supplementary Fig. 7B). Notably, only PD-1, LAG3, and VISTA expression from IHC staining were highly correlated with TIM-3 expression in primary tumors, with the highest positive correlation observed between TIM-3 and PD-1 expression (Supplementary Fig. 7C). The IF further verified the TIM-3 expression within the TME of HGSOC, as TIM-3 was predominantly co-expressed with markers, including CD4, CD8, and CD11c, while minimal expression was observed on pan-cytokeratin (CK) positive tumor cells, indicating that TIM-3 was predominantly expressed on CD4^+^T cells, CD8^+^T cells, and CD11c^+^ myeloid cells rather than tumor cells (Fig. 6B). Flow cytometry was performed to analyze the immune status of T cells and CD11c^+^ myeloid cells in HGSOC patients, and the experimental scheme was displayed in Supplementary Fig. 7D. The gating strategy was displayed in Supplementary Fig. 8A, F. Results showed the high expression and co-expression of TIM-3 and PD-1 on CD4^+^ TILs, CD8^+^TILs, and CD11c^+^ myeloid cells from HGSOC patients (Fig. 6C, D, E). Interestingly, LAG-3 was predominantly co-expressed with TIM-3 on CD8^+^ TILs and was highly expressed on CD4^+^ T cells in both ascites and tumor tissues. CTLA-4 expression was relatively low across CD4^+^ and CD8^+^ T cells from blood, ascites, and tumors, but was co-expressed with TIM-3 on CD4^+^ TILs (Supplementary Fig. 8B–E). Next, we explored the function of CD8^+^TILs and CD11c^+^ myeloid cells in HGSOC patients, and the gating strategy was displayed in Supplementary Fig. 9A, B. TIM-3 and PD-1 double-positive CD8^+^TILs from HGSOC patients exhibited the lowest TNF-α expression, and TIM-3 and PD-1 double-positive CD11c^+^ myeloid cells from HGSOC patients had the lowest IL-12 expression, compared with the single-positive or double-negative ones (Fig. 6F). The ex vivo efficacy of co-blockades of TIM-3 and PD-1 was evaluated using CD8^+^TILs and CD11c^+^ myeloid cells isolated from HGSOC patients, and the gating strategy was displayed in Supplementary Fig. 9C, D. Results indicated that combination therapy could increase TNF-α expression in CD8^+^TILs and IL-12 expression in CD11c^+^ myeloid cells (Fig. 6G, H). In summary, the results from HGSOC patients verified the high co-expression of TIM-3 and PD-1 and efficacy of their co-blockades in enhancing the anti-tumor immunity of CD8^+^TILs and CD11c^+^ myeloid cells.

## Discussion

The limited curative effects of conventional treatments for HGSOC patients are partially attributed to the immunosuppressive TME [25]. Despite advances in immunotherapy, HGSOC patients have shown poor responses to PD-1 blockade monotherapy in clinical trials [5, 6], highlighting the need for alternative therapeutic strategies. Our study identified the important role of TIM-3 in the occurrence of HPD under anti-PD-1 treatment in mouse models and demonstrated the preclinical efficacy of TIM-3 and PD-1 in both in vivo and ex vivo experiments.

HPD is a phenomenon characterized by rapid tumor growth and a poorer prognosis compared to natural disease progression without HPD during ICB therapy [26]. The previous study has identified the female sex as the key clinical indicator for HPD, and thus the high incidence of HPD in gynecological cancer [27]. Similarly, we observed the occurrence of HPD in subcutaneous ID8_VEGF_-bearing mice during anti-PD-1 therapy, as the reduced tumor growth followed by rapid tumor progression, as reported in previous research [28]. Our study showed that anti-TIM-3 and anti-PD-1 therapy could inhibit the occurrence of HPD in a treatment-naïve mouse model and reverse the tumor progression in the established HPD mouse model induced by anti-PD-1 monotherapy. Although the mechanisms underlying the occurrence of HPD are not fully understood, emerging evidence implicates a complex interplay between immune, metabolic, and oncogenic pathways in HPD. CD8^+^T cells, as primary producers of IFN-γ, are recognized to have a paradoxical role in the occurrence and development of HPD [29]. Tumors with active PKM2 and β-catenin signaling pathways are prone to exhibit HPD in the presence of CD8^+^ T cells and IFN-γ [24]. TIM-3 upregulation in CD8^+^TILs has been implicated in HPD in non-small cell lung cancer [30], and our data corroborate this by demonstrating increased TIM-3 expression on CD8^+^TILs and TIDCs of ID8_VEGF_-bearing mice with HPD during anti-PD-1 therapy. Besides the overexpression of immunosuppressive checkpoints, immunosuppressive cell promotion and inflammatory cytokines induced by anti-PD-1 treatment also contribute to HPD. For example, Tregs activation and the release of IFN-γ by anti-PD-1 treatment, suppressing the function of DCs and T cells and promoting the recruitment of MDSCs, lead to HPD [29, 31]. Increased immunosuppressive macrophages and cytokines like IL-10 during PD-1 inhibition treatment contribute to HPD [29]. We also observed the high IL-10 expression in TIDCs from mice with HPD, and TIM-3 and PD-1 co-blockades could effectively decrease the IL-10 secretion and increase IL-12 expression in TIDCs. Moreover, CD8^+^TILs from mice with HPD demonstrated impaired function and high TOX expression, and TIM-3 and PD-1 co-blockades could enhance the anti-tumor immunity of CD8^+^TILs. Interestingly, although we observed the high infiltration of Tregs and MDSCs, minimal effects of combination therapy were shown in the TME of mice with HPD. Collectively, the efficacy of TIM-3 and PD-1 co-blockades in preventing HPD and reversing the tumor progression induced by HPD might rely on enhancing the function of CD8^+^TILs and TIDCs.

Eliciting the full anti-tumor potential of CD8^+^TILs and TIDCs is critical to the success of immunotherapy. Our findings demonstrated the predominant expression and co-expression of TIM-3 and PD-1 on CD8^+^TILs in the TME of HGSOC patients, which was consistent with previous study [17]. Our work further explored the characteristics of DCs in the TME of HGSOC patients, showing the noticeable expression and co-expression of TIM-3 and PD-1 in the TIDCs. Prior research has shown the correlation between TIM-3 and PD-1 expression in CD8^+^TILs, with double-positive ones characterized by poor cytotoxic function, which aligns with findings from prior research [32]. For TIDCs, previous studies showed that the TIM-3 expression inhibits the activation and maturation of DCs, interfering with the ability of endocytosis of extracellular DNA, thus suppressing the anti-tumor immunity in breast cancer [33, 34]. In our study, TIM-3 and PD-1 double-positive TIDCs from HGSOC patients showed the lowest IL-12 production, which indicated that those subsets with impaired anti-tumor function. Findings from our study further depicted the immuno-suppressing impact of TIM-3 and PD-1 in CD8^+^TILs and TIDCs in the TME of HGSOC patients, which showed the potential to be targets for combination therapy.

The alternative inhibitory immune checkpoint upregulation is known for the poor efficacy of ICB, thus, combination therapy is recognized as the therapeutic strategy for overcoming the poor response to single immune checkpoint blockade. The synergistic effects of TIM-3 and PD-1 co-targeted therapy have been reported in previous studies. TIM-3 and PD-1 co-inhibition promote CD8^+^T cells expansion and function in vitro [35, 36]. The dual-inhibition of TIM-3 and PD-1 shows significant anti-tumor effectiveness compared to monotherapy in the HCC xenograft mouse model [37], and enhances the anti-tumor effectiveness of oncolytic virotherapy compared to single blockade in lung cancer [38]. Similarly, our ex vivo experiments using CD8^+^TILs and CD11c^+^ myeloid cells isolated showed that TIM-3 and PD-1 co-targeted therapy could effectively enhance their anti-tumor function, but not their monotherapy. Our study further verified the preclinical efficacy of TIM-3 and PD-1 co-targeted therapy. In the ID8_VEGF_ tumor-bearing mouse model, CD8^+^TILs after combination therapy of TIM-3 and PD-1 inhibitors displayed the highest cytotoxic function and proliferation and the lowest TOX expression, compared with a single inhibitor or isotype; similarly, the maturation, proliferation, and IL-12 production were increased in TIDCs after combination therapy of TIM-3 and PD-1 inhibitors. Although multiple studies have displayed the efficacy of TIM-3 and PD-1 dual blockade approaches, there is limited research on TIM-3 and PD-1 dual blockade approaches in HGSOC. Our experiments in vivo and ex vivo further underscored the preclinical efficacy and immunoregulatory effects of TIM-3 and PD-1 dual blockade in HGSOC.

Despite its contribution, our work had several limitations that warrant further investigation. (1) The cohort included in this study was limited to a relatively small number of tratement-naïve HGSOC patients, and there was a lack of clinical specimens from HGSOC patients with HPD during anti-PD-1 therapy. (2) This study only explored the combinational therapy involving ICB, and further investigation should be done to verify the potential synergies with clinically widely used agents like cisplatin, paclitaxel, or PARP inhibitors. (3) Future studies will focus on the efficacy of anti-TIM-3 monotherapy in preventing HPD and include the clinical samples from HGSOC patients experiencing HPD during anti-PD-1 treatment for direct validation. (4) The markers used to identify the DCs were insufficient, as we defined them as CD11c^+^ myeloid cells instead. (5) The findings from in vivo experiments fully relied on the ID8_VEGF_-bearing mouse model, and other immunocompetent mouse models are required to further validate the generalizability of our conclusions. Furthermore, an intriguing effect of TIM-3/PD-1 co-blockades was observed on tumor-associated macrophages. In a treatment-naïve ovarian tumor-bearing mouse model, upfront combination therapy effectively promoted an anti-tumor microenvironment, as evidenced by an increase in CD86^+^CD206^-^ macrophage cells and a concomitant decrease in CD86^-^CD206^+^ macrophage cells. However, a divergent response was noted in the HPD rescue model, where the same combination therapy led to an expansion of CD86^-^CD206^+^ macrophage cells alongside a reduction in CD86^+^CD206^-^ macrophage cells. We postulate that this discrepancy stems from the profoundly different initial immune landscapes of the two models. In established HPD, the TME is heavily skewed towards immunosuppression. The potent T cell and DC activation induced by the combination therapy may, in this setting, trigger counter-regulatory mechanisms that further expand suppressive CD86^-^CD206^+^ macrophage cells. These findings underscore the remarkable plasticity of the myeloid compartment and suggest that future therapeutic strategies may need to incorporate macrophage-targeting agents, especially when attempting to reverse late-stage HPD.

Our study establishes TIM-3 as a critical mediator of HPD and a promising therapeutic target in HGSOC. TIM-3 and PD-1 co-blockades effectively reverse the exhaustion phenotype of CD8^+^TILs and TIDCs, improving anti-tumor immunity. These preclinical results lay the groundwork for clinical trials investigating TIM-3 and PD-1 co-targeting strategies, offering a new avenue for improving the prognosis of HGSOC patients.

## Materials and methods

### Patients

Sample collection and experimental arrangements for this study received approval from the Institutional Review Board of Tongji Hospital (TJ-IRB20221130) under established guidelines and regulations. Patients enrolled in this research underwent pathological examination before surgery and received no chemotherapy or immunomodulatory drugs. Normal ovaries from participants underwent oophorectomy for reasons other than ovarian pathology and malignancy, and none were treated with chemotherapy or immunosuppressive medications before surgery. Peripheral blood samples were collected from 32 HGSOC patients in EDTA tubes. Fresh tumor tissues from 68 HGSOC patients and ascites from 18 HGSOC patients were collected during the surgery at Tongji Hospital of Tongji Medical College. Supplementary Table 1 presents the clinical and pathological characteristics of the study population.

### IHC and IF

Fresh tissues from HGSOC patients were assembled into a tissue microarray, embedded in paraffin, and then sectioned to 5 μm thickness for subsequent experiments. The tissue sections were then deparaffinized with xylene, and rehydrated, followed by heated antigen retrieval with a heated antigen unmasking solution (Servicebio, G1203). After 1 h of incubation in 5% BSA blocking buffer, primary antibodies were applied overnight at 4 °C. Supplementary Table 2 shows the specific details of antibodies used in the experiment. Anti-human antibodies included TIM-3 (1:100 for IF, 1:250 for IHC)(Cell Signaling Technology, 45208), PD-1 (1:250 for IHC) (Cell Signaling Technology, 86163), LAG-3 (1:250 for IHC) (Abcam, ab209236), CTLA-4 (1:250 for IHC) (Cell Signaling Technology, 53560), TIGIT (1:250 for IHC) (Cell Signaling Technology, 99567), VISTA (1:250 for IHC) (Cell Signaling Technology, 54979), CD11c (1:100 for IF, 1:250 for IHC) (Abcam, ab52632), DC-LAMP (1:400 for IHC) (Novusbio, DDX0191P-100), Anti-human/mouse pan-keratin (1:100 for IF) (Cell Signaling Technology, 4545S), CD4 Monoclonal Antibody (1:100 for IF) (eBioscience, 14-0041-82), and CD8 Monoclonal Antibody (1:100 for IF) (eBioscience, 14-0081-82). For IHC, secondary antibodies were used at a concentration of 1:400 for 1 h at room temperature. For IF, OPAL Multiplex 7-color IHC kit (Akoya Biosciences, NEL871001KT) was used according to the manufacturer’s instruction, followed by incubation with DAPI (Servicebio, GDP1024) for 15 min. For IHC, images were acquired using the HistoFAXS system, and Histoquest software was used to calculate the mean intensity of DAB staining of CD11c and LAMP3 (TissueGnostics, version 7.0.1.165, Vienna, Austria), Image J software (NIH, Bethesda, MD, USA) was used to quantify the DAB staining of TIM-3, PD-1, LAG-3, CTLA-3, VISTA, and TIGIT. For IF, Images were acquired with Operetta CLS high-content imaging system equipment (Perkin Elmer) and PhenoImager HT system (Akoya Biosciences).

### Cell preparation

Peripheral blood mononuclear cells (PBMCs) were obtained via density-gradient centrifugation from fresh blood using Ficoll Paque Premium (Cytiva, 17544202), and isolated leukocytes were obtained from ascites similarly. Isolated leukocytes were prepared from fresh tumor tissues by incubating minced tissue in 100 mg/mL Collagenase Ⅰ and Ⅳ (Servicebio, GC305013, GC305014) at 37 °C with agitation for 1 h and then passed through a 100 μm or 70 μm cell strainers (BD Biosciences, 352350, 352360) were suspended in 40% percoll and then gentled dropped on the 5 mL 70% percoll (Cytiva, 17089102). After 300 *g* centrifugation for 30 min and phosphate-buffered saline (PBS) wash, cells were collected for subsequent analyses.

### Cells culture

Murine OC cell line ID8_VEGF_ was a kind gift from the University of Kansas Medical Center. ID8_VEGF_ were ID8 cells transduced to overexpress vascular endothelial growth factor (VEGF) to recapitulate the high proliferation and invasion of OC in human patients [39], which were cultured in Dulbecco’s modified Eagle’s medium (DMEM) (Gibco, 12100046) with 10% fetal bovine serum in an incubator at 37 °C under a 5% CO_2_ atmosphere.

### Multi-parametric flow cytometry

Supplementary Table 3, and 5 show antibodies and reagents used in the experiment. Fresh PBMCs and mononuclear leukocytes isolated from tumors and ascites from human HGSOC patients or ID8_VEGF_-bearing mice were analyzed using specific antibodies for surface, intercellular, and intranuclear marker expression. FcR Blocker (BioLegend) was used to block the Fc receptors (FcR) for 10 min. Dead cells were excluded using the Zombie NIR Fixable Viability Kit for 30 minutes at room temperature (BioLegend). Cell surface markers, like CD3, CD4, and CD8, were stained with fluorochrome-conjugated antibodies in the dark at 4 °C for 30 min, then cells were washed and resuspended in PBS. For analyzing secretion function, T cells DCs were stimulated using Cell Activation Cocktail (BioLegend) at 37 °C for 4–6 h. Markers for the secretion function of CD8^+^T cells include TNF-α, IFN-γ, Granzyme B, and Perforin, and DCs include IL-12 and IL-10. Among all the markers, intercellular markers, like TNF-α, IFN-γ, and Granzyme B, cells were fixed using the Fixation buffer (BioLegend), and permeabilized using Intracellular Staining Permeabilization Wash Buffer (BioLegend), then stained with fluorochrome-conjugated antibodies in Intracellular Staining Permeabilization Wash Buffer (1×) in the dark at 4 °C for 30 min, then cells were washed and resuspended in PBS. For intranuclear staining, like Ki-67 and thymocyte selection-associated high mobility group box protein (TOX), a Transcription Factor Buffer Set (BD Biosciences) was used per the manufacturer’s instructions. Cells were measured by BECKMAN COULTER CytoFLEX flow cytometer and analyzed using FlowJo software (Ashland).

### Flow cytometry cell sorting

Fresh mononuclear leukocytes isolated from tumors and ascites were obtained as described above. FcR was blocked, and dead cells were excluded, as shown above. Cells were stained with anti-human CD3-PE, anti-human CD8-PC5.5, anti-human CD11c-FITC, and anti-human HLA-DR-APC antibodies. They were sorted into two fluorescence-activated cell sorter tubes to get CD3^+^CD8^+^cells and CD11c^+^HLA-DR^+^cells. All cell sorting was performed using FACS Aria Ⅱ sorter (BD Biosciences, New Jersey). The function of sorted cells was evaluated by flow cytometry as shown above.

### Ex vivo polyclonal T-cell and DCs treatment assay

All mononuclear leukocyte cultures were performed in RPMI 1640 (Gibco, 31800105) supplemented with 10% human serum and 100 μg/mL primocin (InvivoGen, ant-pm-1). Sorted CD3^+^CD8^+^cells or CD11c^+^HLA-DR^+^cells from tumors or ascites were seeded in 24 well plates at 50,000 cells per well. CD3^+^CD8^+^cells were stimulated with the recommended amount of anti-CD3 (BD) and anti-CD28 (BD). CD11c^+^HLA-DR^+^cells were stimulated with the recommended amount of GM-CSF (Novoprotein) and IL-4 (Novoprotein). Blocking anti-human PD-1 antibody, anti-human TIM-3 antibody, and the isotype control antibody were used at 20 μg/mL for 48 h and then evaluated by flow cytometry, as shown above. Antibodies and reagents used are displayed in Supplementary Tables 4, 5.

### Animal studies

Approval for all animal experiments was obtained from the Animal Experiment Ethics Committee of Tongji Hospital (TJH-202210025). Female C57BL/6 mice were purchased from GemPharmatech Co., Ltd (Nanjing), and six-week-old female mice were used for ID8_VEGF_ xenografts. 2 × 10^6^ ID8_VEGF_ cells were injected subcutaneously (s.c.) or intraperitoneally (i.p.). The subcutaneous ID8_VEGF_-bearing model received treatment when the tumor reached 100 mm³ on day 10, and the intraperitoneal model received treatment after IVIS confirmation on day 7. For the subcutaneous mouse model with HPD, anti-PD-1 was given 4 times in total, 200 μg per mouse, i.p., at 3-day intervals. The phenomenon of HPD was defined based on previously published criteria [27]. For the subcutaneous ID8_VEGF_-bearing model, the primary criterion used for defining HPD in this study was TGR. Mice with a more than two-fold increase in the TGR between the pre-treatment period (before the initiation of anti-PD-1 monotherapy, from day 0 to day 10 post-inoculation) and the on-treatment period (during the anti-PD-1 monotherapy, from the start of treatment until observation of rapid tumor growth) were classified as having HPD. The mouse under anti-PD-1 treatment with TGR ≥ 2 was selected and regrouped randomly, followed by being treated with anti-PD-1 monotherapy or anti-PD-1 and anti-TIM-3 combination therapy, 4 times in total, 200 μg per mouse, i.p., at 3-day intervals. For the intraperitoneal mouse model, HPD were defined according to the average radiance. Anti-PD-1 was given 3 times in total, 200 μg per mouse, i.p., at 3-day intervals. IVIS was used to confirm the rapid tumor progression, and those with higher average radiance than the control group were selected and regrouped randomly, followed by being treated with anti-PD-1 monotherapy or anti-PD-1 and anti-TIM-3 combination therapy, 3 times in total, 200 μg per mouse, i.p., at 3-day intervals. For the subcutaneous or intraperitoneal mouse model, isotype, anti-PD-1, and anti-TIM-3 were given at a dose of 200 μg per mouse, i.p., 5 times in total, at 3-day intervals. Tumors from the subcutaneous mouse model were digested with Mouse Tumor Dissociation Kit (Miltenyi), and ascites were obtained from the intraperitoneal mouse model, followed by separating the mononuclear cells from TME using Percoll or Ficoll as described above. For CD4^+^T cells, CD8^+^T cells, and CD11C^+^ cells depletion, mice were injected intraperitoneally with anti-CD4 (clone YTS191), anti-CD8 (clone 53-6.7), and anti-CD11c (clone N418) (Leinco) at a dose of 200 μg per mouse, six times in total. The depletion was verified by flow cytometry after the first and last doses of antibody administration. The sample size for each experiment is indicated as the exact number within the figure legends and represents biological replicates, and were chosen based on the power calculation. Animals were randomly allocated to experimental groups through block randomization, and no animal were excluded from the analysis.

### Quantitative real-time PCR

Total RNA (1 μg) was extracted and cDNA was obtained using FastPure Cell/Tissue Total RNA Isolation Kit V2 and HiScript IV RT SuperMix for qRCR (+gDNA wiper) (all from Vazyme) according to the manufacturer’s instructions. Real-time qPCR was performed using ChamQ Universal SYBR qPCR Master Mix (Vazyme) according to the manufacturer’s instructions for the PCR amplifier (Bio-Rad). The reagents and primers used in this study are shown in Supplementary Tables 5, and 6.

### Statistics analysis

The software for statistical analyses used in this study is displayed in Supplementary Table 7. Normality was tested using the Shapiro-Wilk test. The differences between paired groups were analyzed via paired t-test or Wilcoxon matched-pairs test. The differences between different groups were analyzed via unpaired t-test or Mann-Whitney test. One-way analysis of variance (one-way ANOVA) was performed for multiple group comparisons, and survival curves were compared using the log-rank test. Two factors of correlation were analyzed by Pearson’s correlation for parametric data. Differences were considered to be statistically significant when *P* < 0.05 (*P < 0.05, **P < 0.01, ***P < 0.001, ****P < 0.0001).

## Supplementary information

Supplemental information

## Data Availability

All raw data in this study are available upon reasonable request from the corresponding author.
