# Supplementary material for: Dual blockades of TIM-3 and PD-1 effectively prevent hyper-progression and enhance the efficacy of anti-PD-1 therapy in high-grade serous ovarian cancer
Source: Cell Death Dis. 2025 Nov 28;16(1):867. doi: 10.1038/s41419-025-08231-6 (PMC12663596; doi:10.1038/s41419-025-08231-6)
Supplement: Supplementary file 1 — Supplemental information [file 41419_2025_8231_MOESM1_ESM.docx]

Supplementary Information for

**Dual Blockades of TIM-3 and PD-1 Effectively Prevent Hyper-progression and Enhance the Efficacy of Anti-PD-1 therapy in High-Grade Serous Ovarian Cancer**

Jie Li^1,2#^, Ying Zhou ^1,2#^, Yahan Song^1,2^, Geyang Dai^1,2^, Xi Li^1,2^, Yue Sun^1,2^, Jing Wang^1,2^, Rui Wei^1,2^, Fei Li^1,2*^ and Ling Xi^1,2*^

^#^These two authors contributed equally to this work: Jie Li, Ying Zhou

^*^ **Correspondence**: Fei Li, PhD, Ling Xi, PhD, Department of Obstetrics and Gynecology, National Clinical Research Center for Obstetrics and Gynecology, Tongji Hospital, Tongji Medical College, Huazhong University of Science and Technology, Wuhan, China.

Email: lifei@tjh.tjmu.edu.cn and lxi@tjh.tjmu.edu.cn

**This file includes:**

Supplementary Figure 1-9

Supplementary Table 1-7

**
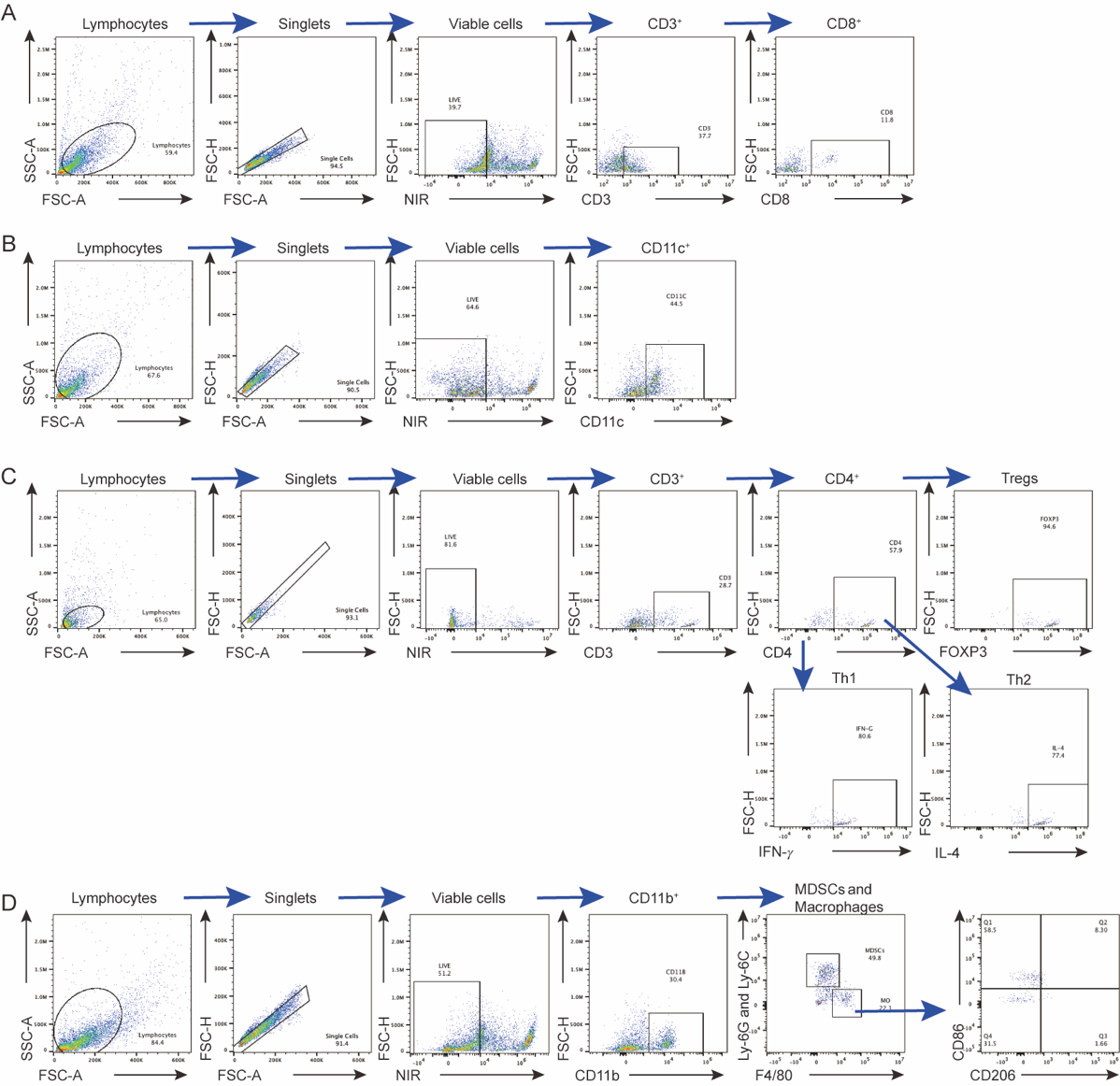
**

**Supplementary Figure 1. The Gating strategy for animal experiments.** (A)The gating strategy of CD8^+^TILs and the percentages of CD3^+^ and CD3^+^CD8^+^ cells. (B) The gating strategy of DCs. (C) The gating strategy of Tregs, Th1 cells, and Th2 cells. (D) The gating strategy of MDSCs, macrophages.

**
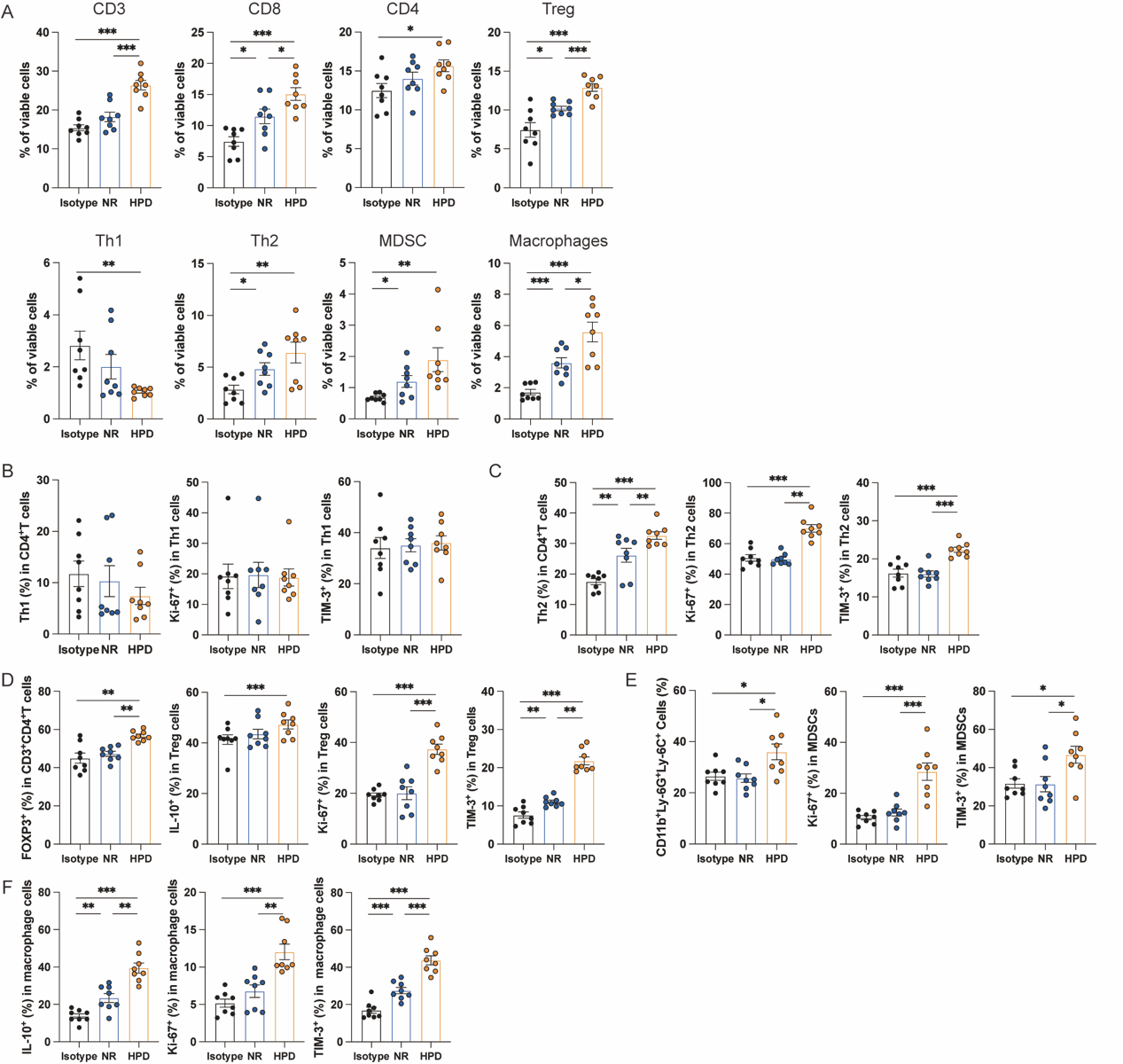
**

**Supplementary Figure 2. Characterization of the function of CD4^+^T cells, MDSCs, and macrophages in ID8_VEGF_-bearing mice demonstrating HPD during anti-PD-1 treatment.**

(A) Percentages of immune cells in total live cells in the TME of mice with HPD. (B-D) Percentages of Th1, Th2 cells, Tregs, TIM-3, and Ki-67 expression in Th1, Th2 cells, and IL-10, Ki-67, and TIM-3 expression in Tregs of tumors from ID8_VEGF_ tumor-bearing mice (n=8 for each group). (E) Percentages of MDSCs, and Ki-67 and TIM-3 expression in MDSCs of tumors from ID8_VEGF_ tumor-bearing mice (n=8 for each group). (F) Percentages of Ki-67 and TIM-3 expression in macrophages from tumors of ID8_VEGF_ tumor-bearing mice (n=8 for each group). Bars represent mean ± SEM, and dots represent individual mice. **P*<0.05, *****P*<0.0001.

**
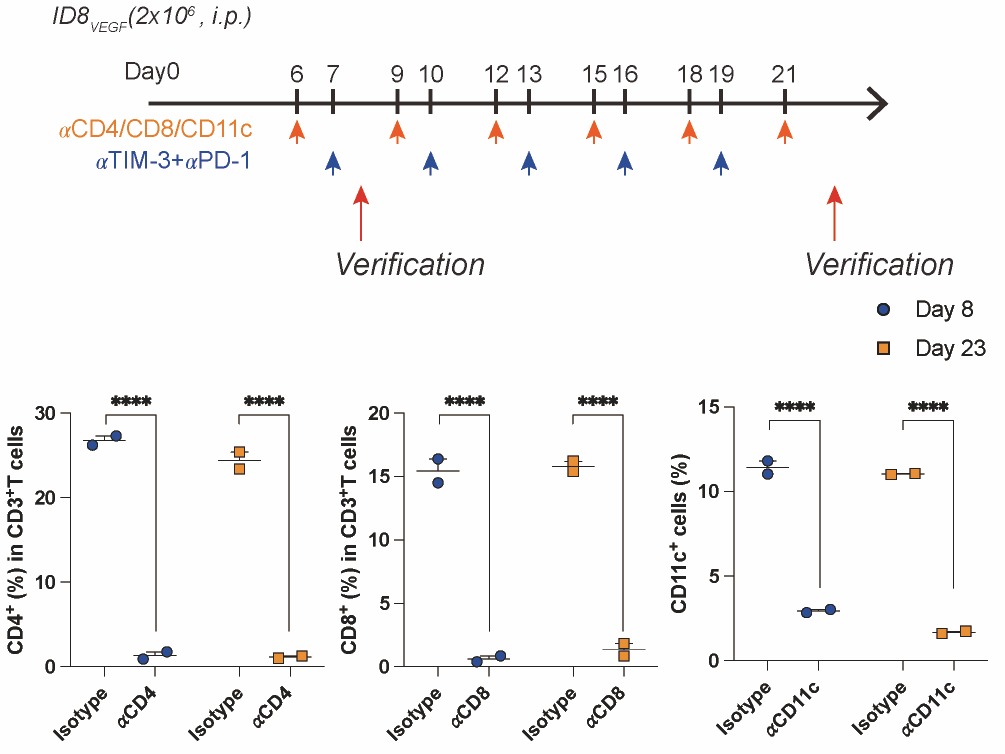
**

**Supplementary Figure 3. The verification of CD4^+^ T cells, CD8^+^ T cells, and CD11c^+^ cells depletion.**


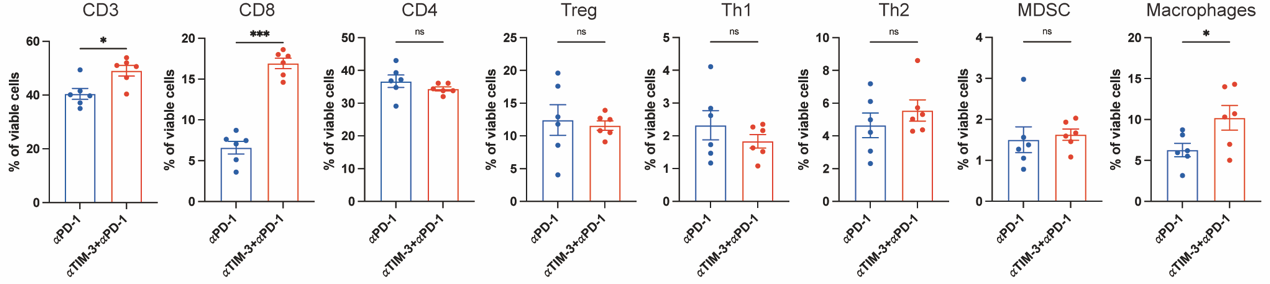


**Supplementary Figure 4.** **Percentages of immune cells in total live cells from mice with HPD after therapy.**

**
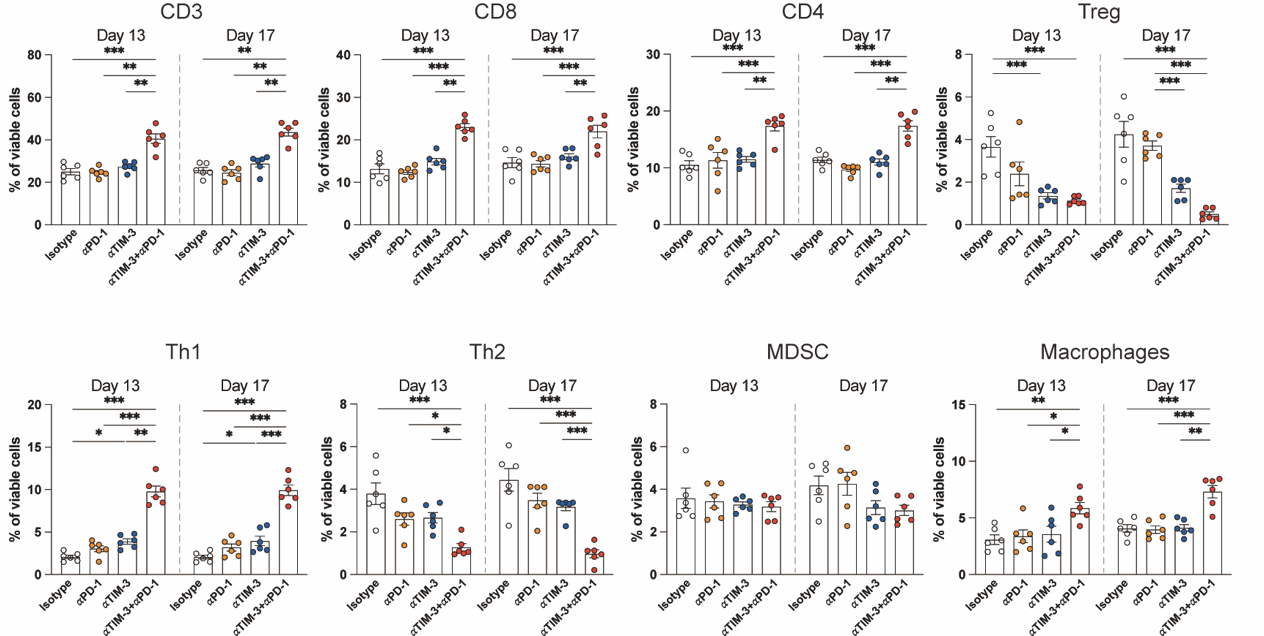
**

**Supplementary Figure 5.** **Percentages of immune cells in total live cells from TME of mice after PD-1 and TIM-3 co-blockades.**

**
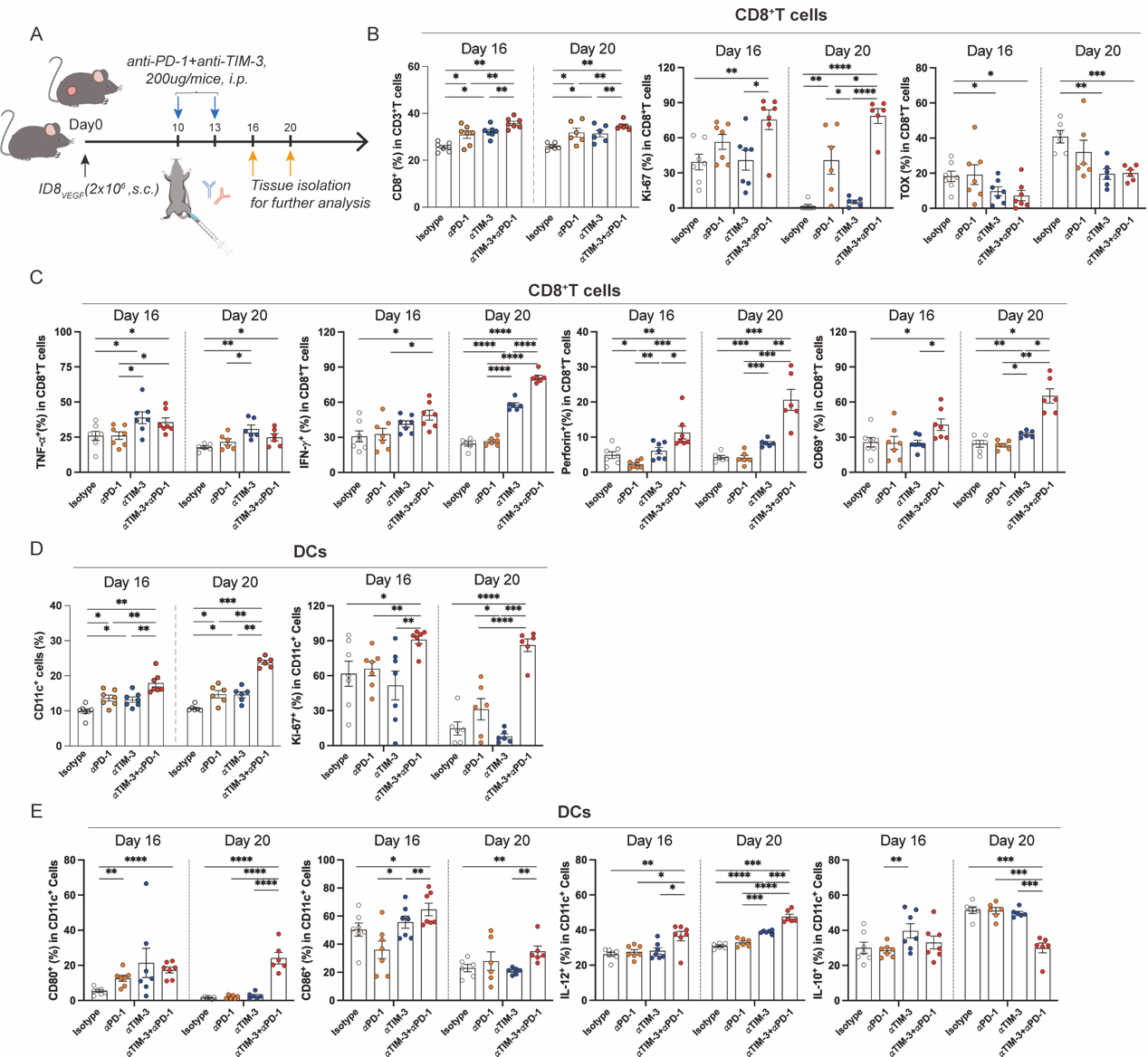
**

**Supplementary Figure 6. TIM-3 and PD-1 co-blockades effectively restore the function and proliferation of CD8^+^TILs and TIDCs from subcutaneous ID8_VEGF_-bearing mice.**

(A) The experimental arrangement and drug administration schedule are displayed. (B and C) Percentages of CD8^+^TILs, and IFN-𝛾, TNF-𝛼, Perforin, CD69, Ki-67, and TOX expression on CD8^+^TILs from ID8_VEGF_ tumor-bearing mice with different treatments on day 16 (n=7) and day 20 (n=6). (D and E) Percentages of TIDCs, and Ki-67, CD80, CD86, IL-12, and IL-10 expression on TIDCs from ID8_VEGF_ tumor-bearing mice with different treatments on day 16 (n=7) and day 20 (n=6). Bars represent mean ± SEM, and dots represent individual mice. **P*<0.05, ***P*<0.01, ****P*<0.001, *****P*<0.0001.

**
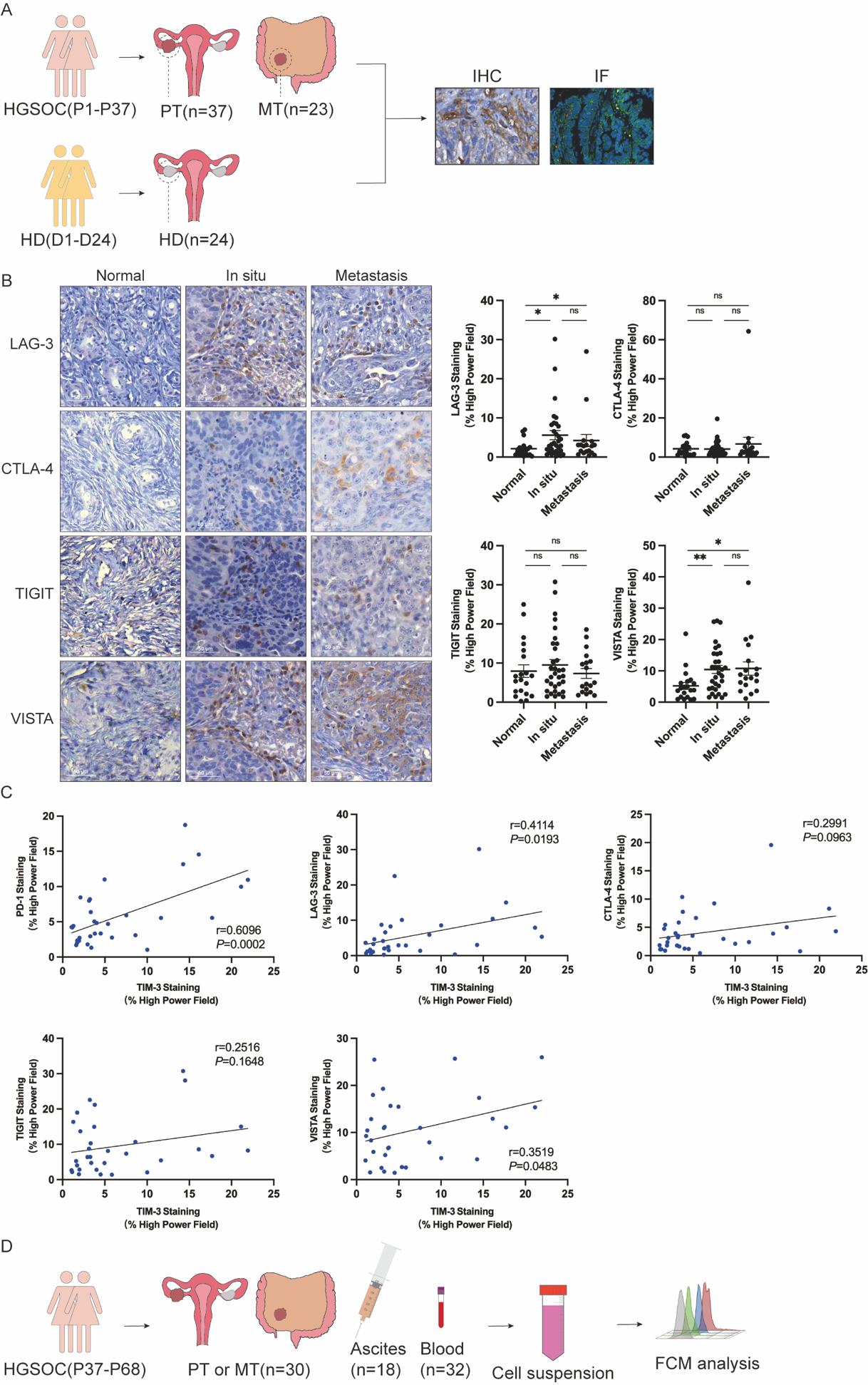
**

**Supplementary Figure 7. The IHC staining of TIM-3 expression is highly correlated with PD-1 expression in tumors of HGSOC patients.**

(A) The experimental scheme for IHC and IF experiments from HGSOC patients (n=37). (B) Representative IHC staining images demonstrate LAG-3, CTLA-4, TIGIT, and VISTA expression and their percentages in normal ovarian tissue (n=24), PT (n=37), and MT (n=23). Scale bars are provided on each image. (C) Correlation between TIM-3 expression and PD-1, LAG-3, CTLA-4, TIGIT, and VISTA expression from IHC staining of tumors in situ from 37 HGSOC patients by Spearman’s correlation analysis. (D) The experimental scheme for flow cytometry (FCM) analysis from HGSOC patients (n=32). Bars represent mean ± SEM, and dots represent the individual patient. *P<0.05, **P<0.01, and ns stands for not significant.

**
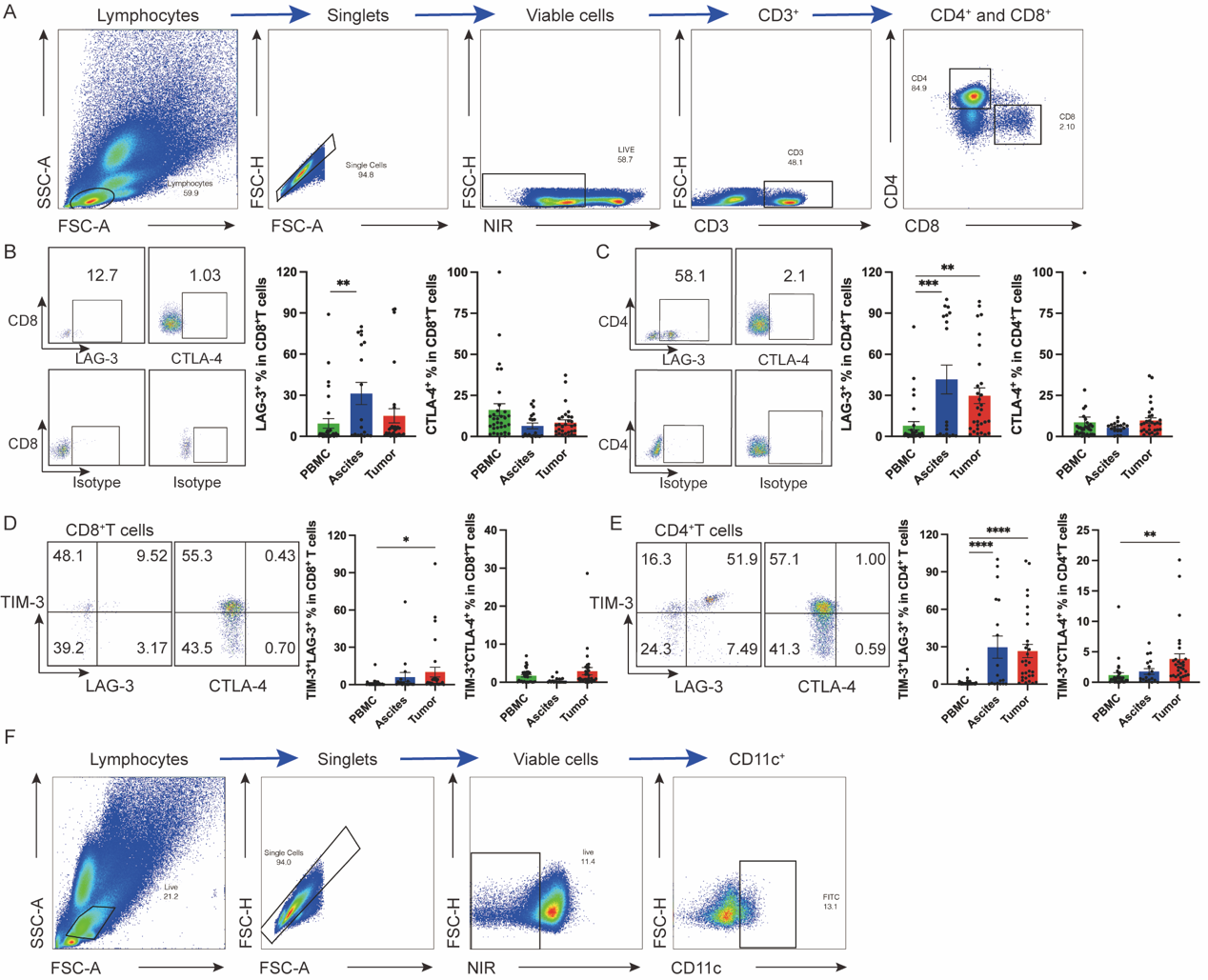
**

**Supplementary Figure 8. LAG-3 is highly expressed in CD4^+^TILs from HGSOC patients.**

(A) Gating scheme for CD8^+^T cells and CD4^+^T cells. (B and C) Representative dot plots and the percentages of LAG-3, and CTLA-4 expression on CD8^+^T cells and CD4^+^T cells in tumors (n=30), ascites (n=18), and blood (n=32) from HGSOC patients. (D and E) Representative dot plots and the percentages of TIM-3 and LAG-3 or CTLA-4 co-expression on CD8^+^T cells and CD4^+^T cells in tumors (n=30), ascites (n=18), and blood (n=32) from HGSOC patients. (F) Gating strategy of CD11c^+^ cells. Bars represent mean ± SEM, and dots represent the individual patient. *P<0.05, **P<0.01, ***P<0.001, ****P<0.0001.

**
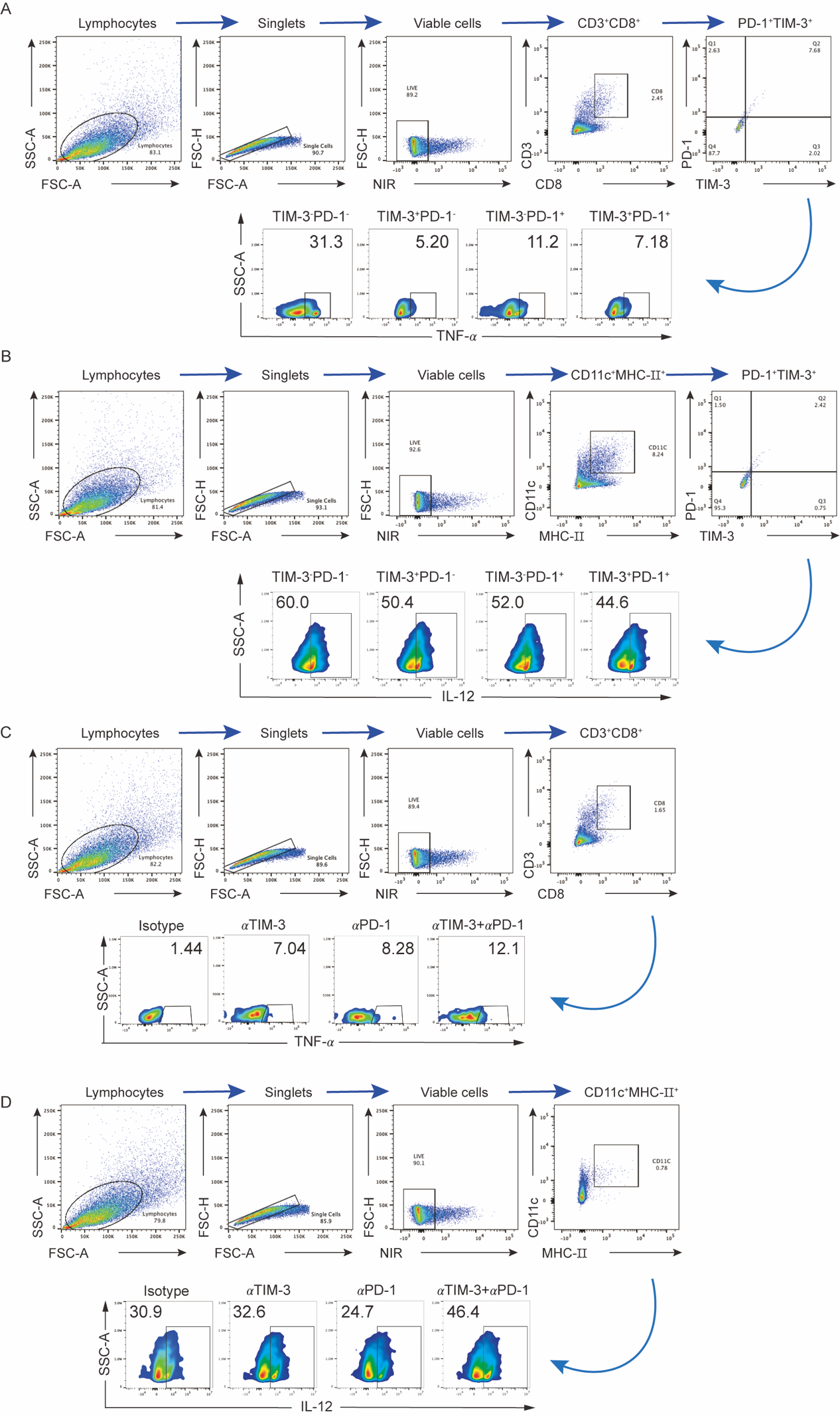
**

**Supplementary Figure 9. Gating strategy for cells from tumors of HGSOC patients.**

(A) The gating strategy for TIM-3^+^PD-1^+^CD8^+^T cells and TNF-𝛼 expression. (B) The gating strategy for TIM-3^+^PD-1^+^CD11c^+^ myeloid cells and IL-12 expression. (C) Gating strategy of CD8^+^TILs from HGSOC patients and TNF-𝛼 expression. (D) Gating strategy of CD11c^+^ myeloid cells from HGSOC patients and IL-12 expression.

**Supplementary Table 1. Characteristics of HGSC patients**

| Variable | Overall cohort (n=68) |
| --- | --- |
| Age |  |
| Mean age(y)±SEM | 55.69±1.37 |
| Stage |  |
| Ⅰ | 2 |
| Ⅱ | 7 |
| Ⅲ | 39 |
| Ⅳ | 20 |
| Debulking |  |
| R0 | 48 |
| R1 | 13 |
| R2 | 7 |

**Supplementary Table 2. Antibodies used in IHC and IF.**

| REAGENT RESOURCE | SOURCE | IDENTIFIER |
| --- | --- | --- |
| Anti-human/mouse pan-keratin clone C11 | Cell Signaling Technology | Cat# 4545S; RRID: AB_490860 |
| Anti-human TIM-3 clone D5D5R | Cell Signaling Technology | Cat# 45208; RRID: AB_2716862 |
| Anti-human DC-LAMP clone 1010E1.01 | Novusbio | Cat# DDX0191P-100; RRID: AB_2827532 |
| Anti-human CD11c clone EP1347Y | Abcam | Cat# Ab52632; RRID: AB_2129793 |
| Anti-human PD-1 clone D4W2J | Cell Signaling Technology | Cat# 86163, RRID: AB_2728833 |
| Anti-human LAG-3 clone EPR20261 | Abcam | Cat# ab209236, RRID: AB_2883982 |
| Anti-human CTLA-4 clone E2V1Z | Cell Signaling Technology | Cat# 53560 |
| Anti-human TIGIT clone E5Y1W | Cell Signaling Technology | Cat# 99567, RRID: AB_2922806 |
| Anti-human VISTA clone D5L5T | Cell Signaling Technology | Cat# 54979, RRID: AB_2799474 |
| CD4 Monoclonal Antibody Clone GK1.5 | eBioscience | Cat#14-0041-82; RRID: AB_467063 |
| CD8a Monoclonal Antibody Clone 53-6.7 | eBioscience | Cat#14-0081-82; RRID: AB_467087 |

**Supplementary Table 3. Antibodies used in flow cytometry.**

| REAGENT RESOURCE | SOURCE | IDENTIFIER |
| --- | --- | --- |
| FITC anti-human CD3 clone UCHT1 | Biolegend | Cat# 300406; RRID: AB_314060 |
| PerCP/Cyanine5.5 anti-human CD4 clone RPA-T4 | Biolegend | Cat# 300529; RRID: AB_893328 |
| PerCP/Cyanine5.5 anti-human CD8a clone RPA-T8 | Biolegend | Cat# 301032; RRID: AB_893422 |
| PE anti-human CD3 clone HIT3a | Biolegend | Cat# 300308; RRID: AB_314044 |
| APC anti-human CD8a clone HIT8a | Biolegend | Cat# 300912; RRID: AB_314116 |
| FITC anti-human CD11c clone 3.9 | Biolegend | Cat# 301604; RRID: AB_314174 |
| APC anti-human HLA-DR clone L243 | Biolegend | Cat# 307610; RRID: AB_314688 |
| APC anti-human CD80 clone 2D10 | Biolegend | Cat# 305220; RRID: AB_2076147 |
| PerCP/Cyanine5.5 anti-human CD86 clone BU63 | Biolegend | Cat# 374216; RRID: AB_2734432 |
| Brilliant Violet 421™ anti-human IFN-γ clone 4S.B3 | Biolegend | Cat# 502532; RRID: AB_2561398 |
| PE anti-human TNF-α clone Mab11 | Biolegend | Cat# 502909; RRID: AB_315261 |
| PerCP/Cyanine5.5 anti-human/mouse Granzyme B clone QA16A02 | Biolegend | Cat# 372212; RRID: AB_2728379 |
| PE anti-human IL-12/IL-23 p40 clone C11.5 | Biolegend | Cat# 501807; RRID: AB_315189 |
| Brilliant Violet 421™ anti-human IL-10 clone JES3-9D7 | Biolegend | Cat# 501422; RRID: AB_2632952 |
| PE anti-human CD366 (Tim-3) clone F38-2E2 | Biolegend | Cat# 345006; RRID: AB_2116576 |
| PerCP/Cyanine5.5 anti-human CD279 (PD-1) clone EH12.2H7 | Biolegend | Cat# 329914; RRID: AB_1595461 |
| Brilliant Violet 421™ anti-human CD279 (PD-1) clone EH12.2H7 | Biolegend | Cat# 329919; RRID: AB_10900818 |
| Brilliant Violet 510™ anti-human CD223 (LAG-3) clone 11C3C65 | Biolegend | Cat# 369317; RRID: AB_2715780 |
| PE/Cyanine7 anti-human CD152 (CTLA-4) clone BNI3 | Biolegend | Cat# 369614; RRID: AB_2632876 |
| PE/Cyanine7 anti-human Galectin-9 clone 9M1-3 | Biolegend | Cat# 348916; RRID: AB_2687013 |
| PE/Cyanine7 anti-human Ki-67 clone Ki-67 | Biolegend | Cat# 350526; RRID: AB_2562872 |
| TOX Monoclonal Antibody (TXRX10), PE | eBioscience | Cat# 12-6502-80; RRID: AB_10853657 |
| FITC anti-mouse CD3 clone 17A2 | Biolegend | Cat# 100204; RRID: AB_312661 |
| PC5.5 anti-mouse CD8a clone 53-6.7 | Biolegend | Cat# 100733; RRID: AB_2075239 |
| APC anti-mouse CD8a clone 53-6.7 | Biolegend | Cat# 100712; RRID: AB_312751 |
| Brilliant Violet 421™ anti-mouse CD279 (PD-1) clone 29F.1A12 | Biolegend | Cat# 135218; RRID: AB_2561447 |
| PE anti-mouse CD366 (Tim-3) clone RMT3-23 | Biolegend | Cat# 119704; RRID: AB_345378 |
| PE anti-mouse CD366 (Tim-3) clone B8.2C12 | Biolegend | Cat# 134004; RRID: AB_1626177 |
| TOX Monoclonal Antibody (TXRX10), eFluor 660 | Invitrogen | Cat# 50-6502-82; RRID: AB_2574265 |
| PE/Cy7 anti-mouse Ki-67 clone 16A8 | Biolegend | Cat# 652426; RRID: AB_2632694 |
| PE anti-mouse IFN-𝛾 clone XMG1.2 | Biolegend | Cat# 505808; RRID: AB_315402 |
| Brilliant Violet 421™ anti-mouse TNF-𝛼 clone MP6-XT22 | Biolegend | Cat# 506327; RRID: AB_10900823 |
| FITC anti-mouse CD11c clone N418 | Biolegend | Cat# 117306; RRID: AB_313775 |
| PerCP/Cyanine5.5 anti-mouse CD86 clone GL-1 | Biolegend | Cat# 105028; RRID: AB_2074994 |
| APC anti-mouse CD80 clone 16-10A1 | Biolegend | Cat# 104714; RRID: AB_313135 |
| Brilliant Violet 421™ anti-mouse IL-10 clone JES5-16E3 | Biolegend | Cat# 505022; RRID: AB_2563240 |
| PE anti-mouse IL-12 clone C15.6 | Biolegend | Cat# 505204; RRID: AB_315368 |
| APC anti-mouse IA-IE clone M5/114.15.2 | Biolegend | Cat# 107614; RRID: AB_313329 |
| Brilliant Violet 605™ anti-mouse IL-4 clone 11B11 | Biolegend | Cat# 504126; RRID: AB_2686971 |
| FITC anti-mouse/human CD11b clone M1/70 | Biolegend | Cat# 101205; RRID: AB_312788 |
| Alexa Fluor® 647 Rat Anti-Mouse CD206 | BD Biosciences | Cat# 565250; RRID: AB_2739133 |
| BV510 Rat Anti-Mouse Ly-6G and Ly-6C | BD Biosciences | Cat# 563040; RRID: AB_ 2722496 |
| BV650 Rat Anti-Mouse TNF | BD Biosciences | Cat# 563943; RRID: AB_2738498 |
| PE Anti-Mouse Perforin | eBioscience | Cat# 12-9392-82; RRID: AB_466243 |
| BV510 Hamster Anti-Mouse CD69 | BD Biosciences | Cat# 563030; RRID: AB_2737963 |
| PE Rat Anti-Mouse F4/80 | BD Biosciences | Cat# 565410; RRID: AB_2687527 |
| Brilliant Violet 650™ anti-mouse TIM-3 clone 25F.1D6 | BD Biosciences | Cat# 755162; RRID: AB_3687572 |
| FITC anti-mouse CD11c clone HL3 | BD Biosciences | Cat# 561045; RRID: AB_396683 |
| PC5.5 anti-mouse CD4 clone RM4-5 | BD Biosciences | Cat# 561115; RRID: AB_ 393977 |
| PE anti-mouse CD8a clone 5H10-1 | BD Biosciences | Cat# 567630; RRID: AB_ 2916674 |

**Supplementary Table 4. Antibodies used in treatment in vivo and invitro.**

| REAGENT RESOURCE | SOURCE | IDENTIFIER |
| --- | --- | --- |
| Rat anti trinitrophenol Isotype Control (IgG2a) | BioXCell | Cat# BE0089; RRID: AB_1107769 |
| Anti-mouse TIM-3 clone RMT3-23 | BioXCell | Cat# BE0115; RRID: AB_10949464 |
| Anti-mouse PD-1 clone RMP1-14 | BioXCell | Cat# BE0146; RRID: AB_10949053 |
| Rat IgG kappa Isotype Control | eBioscience | Cat# 16-4301-81; RRID: AB_470153 |
| Ultra-LEAF^TM^ Purified anti-human CD366 (Tim-3) clone F38-2E2 | Biolegend | Cat# 345010; RRID: AB_11150780 |
| Ultra-LEAF^TM^ Purified anti-human CD279 (PD-1) clone EH12.2H7 | Biolegend | Cat# 329926; RRID: AB_11150780 |
| Anti-mouse CD4 | Leinco | Cat# C3220; RRID: AB_ 2893558 |
| Anti-mouse CD8a | Leinco | Cat# C375; RRID: AB_ 2737478 |
| Anti-mouse CD11c | Leinco | Cat# C2119; RRID: AB_ 2737458 |

**Supplementary Table 5. Reagents used in the research.**

| REAGENT RESOURCE | SOURCE | IDENTIFIER |
| --- | --- | --- |
| Anti-human CD3 clone HIT3𝛼 | BD Biosciences | Cat# 555336; RRID: AB_395742 |
| Anti-human CD28 clone CD28.2 | BD Biosciences | Cat# 555725; RRID: AB_396068 |
| Primocin | InvivoGen | Cat# ant-pm-1 |
| Recombinant human IL4 | Novoprotein | Cat# C50 |
| Recombinant human IL-2 | Peprotech | Cat# 20002 |
| GMCSF | Novoprotein | Cat# C003 |
| Zombie NIR Fixable Viability Kit | Biolegend | Cat# 423105 |
| TrueStain FcX Block | Biolegend | Cat# 422302; RRID: AB_2818986 |
| TruStain FcX (anti-mouse CD16/32) | Biolegend | Cat# 101320; RRID: AB_1574975 |
| Cell Activation Cocktail | Biolegend | Cat# 423303 |
| Fixation buffer | Biolegend | Cat# 420801 |
| Intracellular Staining Permeabilization Wash Buffer (10X) | Biolegend | Cat# 421002 |
| Transcription Factor Buffer Set | BD Biosciences | Cat# 562574; RRID: AB_2869424 |
| Matrigel | Corning | Cat# 354234 |
| GolgiStop | BD Biosciences | Cat# 554724 |
| Mouse Tumor Dissociation Kit | Miltenyi | Cat# 130-096-730 |
| FastPure Cell/Tissue Total RNA Isolation Kit V2 | Vazyme | Cat# RC112 |
| **HiScript IV RT SuperMix for qRCR (+gDNA wiper)** | Vazyme | Cat# R423-01 |
| **ChamQ Universal SYBR qPCR Master Mix** | Vazyme | Cat# Q711-02 |

**Supplementary Table 6. Primer sequences used in quantitative RT-PCR.**

| Gene name | Primer sequence |
| --- | --- |
| *Havcr2* | Forward：5’ TCAGGTCTTACCCTCAACTGTG 3’  Reverse：5’ GGGCAGATAGGCATTTTTACCA 3’ |
| *Lag3* | Forward：5’ CTGGGACTGCTTTGGGAAG 3’  Reverse：5’ GGTTGATGTTGCCAGATAACCC 3’ |
| *Ctla4* | Forward：5’ TTTTGTAGCCCTGCTCACTCT 3’  Reverse：5’ CTGAAGGTTGGGTCACCTGTA 3’ |
| *Tigit* | Forward：5’ GAATGGAACCTGAGGAGTCTCT 3’  Reverse：5’ AGCAATGAAGCTCTCTAGGCT 3’ |
| *Vsir* | Forward：5’ GGAACCCTGCTCCTTGCTATT 3’  Reverse：5’ TTGTAGATGGTCACATCGTGC 3’ |
| *Cd274* | Forward：5’ GCTCCAAAGGACTTGTACGTG 3’  Reverse：5’ TGATCTGAAGGGCAGCATTTC 3’ |
| *Pvr* | Forward：5’ GGAACCCTGCTCCTTGCTATT 3’  Reverse：5’ TTGTAGATGGTCACATCGTGC 3’ |
| *Cd4* | Forward：5’ TCCTAGCTGTCACTCAAGGGA 3’  Reverse：5’ TCAGAGAACTTCCAGGTGAAGA 3’ |
| *Cd8* | Forward：5’ CCGTTGACCCGCTTTCTGT 3’  Reverse：5’ CGGCGTCCATTTTCTTTGGAA 3’ |
| *Cd19* | Forward：5’ GGAGGCAATGTTGTGCTGC 3’  Reverse：5’ ACAATCACTAGCAAGATGCCC 3’ |
| *Itgax* | Forward：5’ CTGGATAGCCTTTCTTCTGCTG 3’  Reverse：5’ GCACACTGTGTCCGAACTCA 3’ |
| *Cd56* | Forward：5’ GACAGAACCCGAAAAGGGC 3’  Reverse：5’ GTTGGGGACCGTCTTGACTT 3’ |
| *Nos* | Forward：5’ GTTCTCAGCCCAACAATACAAGA 3’  Reverse：5’ GTGGACGGGTCGATGTCAC 3’ |
| *Cd163* | Forward：5’ ATGGGTGGACACAGAATGGTT 3’  Reverse：5’ CAGGAGCGTTAGTGACAGCAG 3’ |
| *Gzmb* | Forward：5’ CCACTCTCGACCCTACATGG 3’  Reverse：5’ GGCCCCCAAAGTGACATTTATT 3’ |
| *Il10* | Forward：5’ GCTCTTACTGACTGGCATGAG 3’  Reverse：5’ CGCAGCTCTAGGAGCATGTG 3’ |
| *Il12* | Forward：5’ TGGTTTGCCATCGTTTTGCTG 3’  Reverse：5’ ACAGGTGAGGTTCACTGTTTCT 3’ |
| *Gapdh* | Forward：5’ AGGTCGGTGTGAACGGATTTG 3’  Reverse：5’ TGTAGACCATGTAGTTGAGGTCA 3’ |

**Supplementary Table 7. Software used in the research.**

| Software and Algorithms | company | website |
| --- | --- | --- |
| FlowJo Version 10 | FlowJo LLC | [https://www.flowjo.com](https://www.flowjo.com/) |
| Prism Version 9 | GraphPad | <https://www.graphpad.com/scientific-software/prism/> |
| Histoquest version 7.0.1.165 | TissueGnostics | <https://tissuegnostics.com/> |
| **Image J version 1.8.0** | National Institutes of Health | <https://imagej.net/ij/> |
| Living Image 4.5.5 software | **PerkinElmer** | https://www.perkinelmer.com.cn |
